# Exogenous nitrogen mitigation of cadmium stress in *Pinellia ternata* is associated with the regulation of arginine and proline metabolism

**DOI:** 10.3389/fpls.2026.1900436

**Published:** 2026-09-21

**Authors:** Zhigui He, Yuzhen Dai, Qing Zhang

**Affiliations:** 1 School of Food and Health, Guilin Tourism University, Guilin, China; 2 College of Marine Science and Biotechnology, Guangxi Minzu University, Nanning, China; 3 School of Environmental Science and Engineering, Guilin University of Technology, Guilin, China

**Keywords:** cadmium stress, metabolomics, nitrogen supplementation, *Pinellia ternata*, transcriptomics

## Abstract

Cadmium (Cd) contamination is a major constraint limiting the yield and quality of medicinal plants. In this study, the effects of exogenous nitrogen on alleviating Cd toxicity in *Pinellia ternata* seedlings were investigated, and the underlying mechanisms were explored using integrated physiological, transcriptomic, untargeted metabolomic, and rhizosphere microbial analyses. Nitrogen supplementation (150 mg N kg^-^¹ supplied as urea) mitigated Cd-induced growth inhibition, increasing root length by approximately 31% on days 6 and 9 and partially improving leaf area by day 9. Nitrogen supplementation also significantly decreased the root-to-shoot Cd translocation factor at all three sampling times, while root Cd concentrations remained largely unchanged; shoot Cd concentration was significantly reduced on day 9. Physiologically, nitrogen supplementation modulated antioxidant enzyme activity and reduced malondialdehyde (MDA) accumulation at the early stage, consistent with a time-dependent alleviation of membrane lipid peroxidation under Cd stress. Integrated multi-omics analyses revealed extensive transcriptional and metabolic reprogramming. Transcriptomic analysis identified 36,036, 9,373, and 33,136 differentially expressed genes on days 3, 6, and 9, respectively, whereas metabolomic profiling identified 947, 1,203, and 729 differentially accumulated metabolites, respectively. KEGG enrichment analysis of the differentially accumulated metabolites consistently highlighted arginine and proline metabolism across the three sampling times, accompanied by enrichment of related pathways, including ascorbate and aldarate metabolism, glutathione metabolism, ABC transporters, and nitrogen metabolism. Within the arginine and proline metabolism pathway, genes encoding pyrroline-5-carboxylate reductase, spermidine synthase, and aldehyde dehydrogenase family members showed treatment-dependent differential expression, accompanied by concurrent changes in the relative abundances of proline, glutamine, and polyamines. Collectively, these findings indicate that exogenous nitrogen is associated with improved Cd tolerance in *P. ternata* through coordinated regulation of Cd translocation, arginine and proline metabolism, antioxidant and transport-related processes, and rhizosphere-associated responses. This study provides integrated evidence for nitrogen-mediated Cd tolerance and offers insights into nutrient management for mitigating Cd stress during *P. ternata* cultivation.

## 1 Introduction

Cadmium (Cd) is one of the most phytotoxic and persistent heavy metals in agricultural soils and mainly originates from mining, industrial emissions, phosphate fertilizer overuse, and wastewater irrigation (Hu et al., 2025; Dong et al., 2026). Because of its high mobility and bioaccumulation potential, Cd is readily taken up by plant roots, leading to oxidative stress, inhibited photosynthesis and root development, disrupted nutrient homeostasis, and ultimately reduced biomass and quality (Yu et al., 2025; Fan et al., 2023). Cd contamination is particularly concerning for medicinal plants because it not only reduces yield but also threatens the safety of medicinal materials (Al-Khayri et al., 2023). *Pinellia ternata* (Thunb.) Breit., a perennial herbaceous plant of the Araceae family, is an important traditional Chinese medicinal herb widely used for its antitussive, antiemetic, anti-inflammatory, and expectorant properties (Zhang et al., 2025). However, *P. ternata* is sensitive to abiotic stresses, and Cd contamination in cultivation areas may severely impair plant growth, tuber quality, and medicinal safety (Ng et al., 2023).

Cd phytotoxicity is largely associated with the overproduction of reactive oxygen species (ROS), which causes lipid peroxidation, malondialdehyde (MDA) accumulation, inhibition of antioxidant enzymes, damage to the photosynthetic apparatus, and disturbance of nitrogen and carbon metabolism (Balestrasse et al., 2005; Yang et al., 2020). Therefore, effective and low-cost agronomic strategies are urgently needed to improve Cd tolerance in medicinal plants. Nitrogen (N), an essential macronutrient, plays important roles in plant growth and stress responses. An adequate N supply can alleviate Cd toxicity by enhancing antioxidant defense, promoting the synthesis of chelating compounds such as glutathione and metallothioneins, regulating osmotic adjustment, and reprogramming primary and secondary metabolic pathways (Yang et al., 2020; He et al., 2021; Zhang et al., 2024). Among these processes, arginine and proline metabolism is particularly important because proline functions as an osmoprotectant, ROS scavenger, and protein stabilizer, whereas arginine-derived polyamines contribute to membrane stabilization, Cd chelation, and stress signaling (Meena et al., 2019; Spormann et al., 2023).

Although increasing evidence indicates that nitrogen application can improve heavy metal tolerance in food crops and model plants, the mechanisms underlying N-mediated Cd tolerance in medicinal plants remain insufficiently understood. Previous studies on *P. ternata* have focused mainly on responses to individual abiotic stresses, such as heat, drought, antibiotics, and microplastics, using physiological or transcriptomic approaches (Guo et al., 2022; Duan et al., 2025; Zhang et al., 2025; Bo et al., 2024; Ji et al., 2025). However, few studies have systematically investigated the interaction between exogenous nitrogen and Cd stress in *P. ternata*. In particular, whether arginine and proline metabolism act as core regulatory pathways linking antioxidant defense, metabolic reprogramming, and rhizosphere microbial stability under N supplementation and Cd stress remains unclear.

In this study, morphological and physiological measurements were integrated with transcriptomic, metabolomic, and rhizosphere microbial community analyses to investigate the alleviating effects of exogenous nitrogen on Cd toxicity in *P. ternata* seedlings. The objectives were to (1) evaluate the effects of nitrogen supplementation on Cd-induced growth inhibition, oxidative damage, and Cd accumulation and translocation; (2) elucidate the underlying molecular and metabolic mechanisms, with a particular emphasis on arginine and proline metabolism. This study provides integrated multi-omics evidence for N-mediated Cd tolerance in a medicinal plant and offers insights into nutrient management for mitigating Cd stress during *P. ternata* cultivation.

## 2 Materials and methods

### 2.1 Plant materials and growth conditions

Uniform tissue-cultured seedlings of *P. ternata* (Thunb.) Breit.were selected as the experimental materials. After surface sterilization with a 0.1% (w/v) HgCl_2_ solution for 5 min and thorough rinsing with sterile distilled water, the seedlings were transplanted into plastic pots containing sterilized cultivation substrate. The substrate consisted of a mixture of peat:vermiculite:perlite (3:1:1, v/v/v) adjusted to a pH of 6.0 and autoclaved at 121 °C for 30 min. Plants were grown in a controlled-environment growth chamber with a 14 h/10 h light/dark photoperiod, a photosynthetic photon flux density of 300 μmol m^-^² s^-^¹ (provided by LED lamps), a day/night temperature of 25 °C/18 °C, and a relative humidity of 70%. Substrate moisture content was maintained at 60% of the water-holding capacity (WHC) using a time-domain reflectometry system (Chen et al., 2025; Zhang et al., 2025).

### 2.2 Experimental treatments

Four treatments with six biological replicates each were established in a completely randomized design: CK, control without nitrogen or Cd addition; N, 150 mg N kg^-^¹ supplied as urea [CO(NH_2_)_2_; equivalent to approximately 326 mg urea kg^-^¹ substrate]; Cd, 50 μM CdCl_2_; and N+Cd, 150 mg N kg^-^¹ supplied as urea + 50 μM CdCl_2_.

Urea was dissolved in deionized water and applied to each pot by drenching the substrate surface at a rate equivalent to 150 mg N kg^-^¹ substrate (approximately 326 mg urea kg^-^¹ substrate), a dose selected on the basis of previous studies on nitrogen supplementation in heavy-metal-stressed plants (Zhang et al., 2024). CdCl_2_ was dissolved in deionized water to prepare a 50 μM stock solution, which was subsequently applied to each pot by root drenching. Both reagents were applied on day 0 at the initiation of the experiment, and the treatments were maintained continuously for 9 days.

The Cd concentration (50 μM CdCl_2_) was selected on the basis of preliminary experiments as a sublethal dose that induced clear stress symptoms, including growth inhibition and oxidative damage, while sufficient plant viability was maintained for multiple time-point physiological and multiomics analyses. Treatments were applied continuously for 9 days. Samples for physiological analyses were collected on days 3, 6, and 9. Root tips for transcriptomic and metabolomic analyses and rhizosphere soil samples for 16S rRNA gene sequencing were collected at the same three time points and immediately frozen in liquid nitrogen before being stored at −80 °C (Yang et al., 2020; Zhang et al., 2024).

All six biological replicates were used for growth and physiological measurements. For transcriptomic, metabolomic, and rhizosphere microbial analyses, three independent biological replicates were randomly selected from each treatment at each sampling time. Each biological replicate consisted of one independently grown plant/pot, and no technical replicates were treated as biological replicates. In total, 36 samples were used for transcriptomic analysis, 36 samples for metabolomic analysis, and 36 rhizosphere soil samples for 16S rRNA gene sequencing, corresponding to 4 treatments × 3 sampling times × 3 biological replicates.

### 2.3 Growth parameter measurements

At each sampling time, the plants were harvested and separated into roots and shoots. Root morphology (total root length) was analyzed using a root scanner (WinRHIZO, Regent Instruments, Canada) and ImageJ software. The leaf area was determined using ImageJ. The fresh weights of different organs were recorded immediately, and the organs were then oven-dried at 65 °C for 48 h to obtain dry weights. Biomass is expressed as dry weight per plant (Ng et al., 2023; Zhang et al., 2025).

### 2.4 Determination of Cd concentrations and translocation factor

Root and shoot samples were oven-dried, ground into powder, and digested with HNO_3_-H_2_O_2_ solution. Cd concentrations were determined using ICP-MS and expressed as mg kg^-^¹ DW. The root-to-shoot translocation factor (TF) was calculated as follows:


$$\text{T}\text{F}=C_{shoot}/C_{root}$$

where *C_shoot_* and *C_root_* represent the Cd concentrations in shoots and roots, respectively.

### 2.5 Antioxidant enzyme activities and lipid peroxidation

Fresh root samples (0.5 g) were homogenized in ice-cold 50 mM potassium phosphate buffer (pH 7.0) containing 1% (w/v) polyvinylpyrrolidone. The homogenate was centrifuged at 12,000× g for 20 min at 4 °C, and the supernatant was used for the enzyme assays.

Superoxide dismutase (SOD) activity was determined by the nitroblue tetrazolium photoreduction method. Peroxidase (POD) activity was measured using the guaiacol oxidation method. Catalase (CAT) activity was assayed by monitoring the decrease in H_2_O_2_ absorbance at 240 nm. MDA content, which serves as an indirect proxy for membrane lipid peroxidation, was determined using the thiobarbituric acid reaction. All spectrophotometric measurements were performed using a UV–Vis spectrophotometer (Duan et al., 2025; Zhang et al., 2024).

### 2.6 Transcriptome sequencing

Total RNA was extracted from root tip tissues using TRIzol reagent (Invitrogen, USA) according to the manufacturer’s instructions, followed by DNase I digestion to remove genomic DNA. RNA quality and quantity were assessed using a NanoDrop spectrophotometer and an Agilent 2100 Bioanalyzer.

cDNA libraries were prepared from mRNA using the NEBNext Ultra II RNA Library Prep Kit for Illumina (New England Biolabs, USA). Sequencing was performed on an Illumina NovaSeq 6000 platform with 150 bp paired-end reads, targeting approximately 50 million reads per sample.

Raw reads were processed to obtain clean data by removing adapters, poly-N sequences and low-quality reads. *De novo* transcriptome assembly was performed using Trinity with default parameters. Clean reads were mapped to the *P. ternata* reference transcriptome using HISAT2. Differential expression analysis was performed using the DESeq2 R package with the criteria |log_2_FC| > 1 and a false discovery rate (FDR) < 0.05. Functional annotation and KEGG pathway enrichment analyses of differentially expressed genes (DEGs) were conducted using the Kyoto Encyclopedia of Genes and Genomes (KEGG) database, with a particular focus on heavy metal detoxification, nitrogen metabolism and amino acid metabolism pathways (Guo et al., 2022; Ji et al., 2025).

### 2.7 Metabolome analysis

The root samples were freeze-dried and ground into a powder. Metabolites were extracted with 80% (v/v) methanol by vortexing and centrifugation at 4 °C. The supernatant was filtered and analyzed by ultrahigh-performance liquid chromatography coupled with quadrupole time-of-flight mass spectrometry (UHPLC–QTOF–MS) (Agilent 1290/6545XT, USA).

Chromatographic separation was performed on a ZORBAX Eclipse Plus C18 column (2.1 × 100 mm, 1.8 μm) using a suitable gradient eluent. Mass spectra were acquired in both positive and negative electrospray ionization (ESI) modes over a m/z range of 50–1000. Metabolites were annotated against the HMDB, KEGG, and an in-house *P. ternata* metabolite database (Duan et al., 2025; Zhang et al., 2025). Signal intensities of key metabolites annotated to the arginine and proline metabolism pathway were extracted and used for semi-quantitative comparison between treatments.

### 2.8 Rhizosphere soil DNA extraction and 16S rRNA gene sequencing

Rhizosphere soil samples were collected on days 3, 6, and 9 using the method described by Lundberg et al. (2012). Briefly, roots with tightly adhering soil were gently shaken to remove loose soil, and the remaining rhizosphere soil was brushed off and immediately frozen in liquid nitrogen.

Total genomic DNA was extracted from 0.5 g of rhizosphere soil using the FastDNA™ Spin Kit for Soil (MP Biomedicals, USA) following the manufacturer’s instructions. DNA quality and concentration were assessed using a NanoDrop 2000 spectrophotometer (Thermo Scientific, USA) and 1% agarose gel electrophoresis.

The V3-V4 hypervariable region of the bacterial 16S rRNA gene was amplified with the primer pair 343F (5′-TACGGRAGGCAGCAG-3′) and 798R (5′-AGGGTATCTAATCCT-3′) (Nossa et al., 2010). PCR products were purified, quantified, and pooled in equimolar amounts. Libraries were prepared with the TruSeq^®^ DNA PCR-Free Sample Preparation Kit (Illumina, USA) and sequenced on an Illumina NovaSeq 6000 platform to produce 250 bp paired-end reads.

Raw reads were processed using QIIME 2 (Bolyen et al., 2019). Quality filtering, denoising, and chimera removal were performed with DADA2 to generate amplicon sequence variants (ASVs). Taxonomy was assigned using the SILVA 138 database. Alpha diversity (ACE index) and community composition were analyzed to assess the effects of treatment on rhizosphere microbial communities.

Beta diversity was assessed by non-metric multidimensional scaling (NMDS) based on Bray–Curtis dissimilarities. Functional potential was predicted with PICRUSt2 against the COG database, and differences in predicted COG abundances between treatments were tested by Welch’s t-test (*P* < 0.05).

### 2.9 Statistical analysis

Growth, physiological, Cd concentration, and translocation factor data are presented as means ± SEs. Differences among treatments at each sampling time point were analyzed by one-way analysis of variance (ANOVA), followed by Duncan’s multiple range test at P < 0.05. Differential expression, metabolite screening, and microbial community analyses were performed using the criteria and workflows described above. Differences in relative metabolite abundances between the Cd and N+Cd treatments were assessed by Student’s t-test (n = 3), and associations between transcript abundance and metabolite levels were evaluated by Spearman rank correlation (*P* < 0.05).

## 3 Results

### 3.1 Effects of exogenous nitrogen on growth parameters under Cd stress

As shown in **Figure 1** and **Supplementary Table 1**, Cd stress significantly inhibited the growth of *P. ternata* seedlings in a time-dependent manner. Representative images of the seedlings at days 3, 6, and 9 (**Figure 1A**) indicate that compared with the control (CK), Cd-treated plants displayed stunted growth; shorter and thinner roots; smaller leaves; and reduced overall vigor. In contrast, the plants in the N treatment group maintained a morphology similar to that in the CK treatment group, whereas the N+Cd treatment clearly alleviated Cd-induced growth inhibition, resulting in taller plants and better-developed root systems, particularly at days 6 and 9.

**Figure 1:**
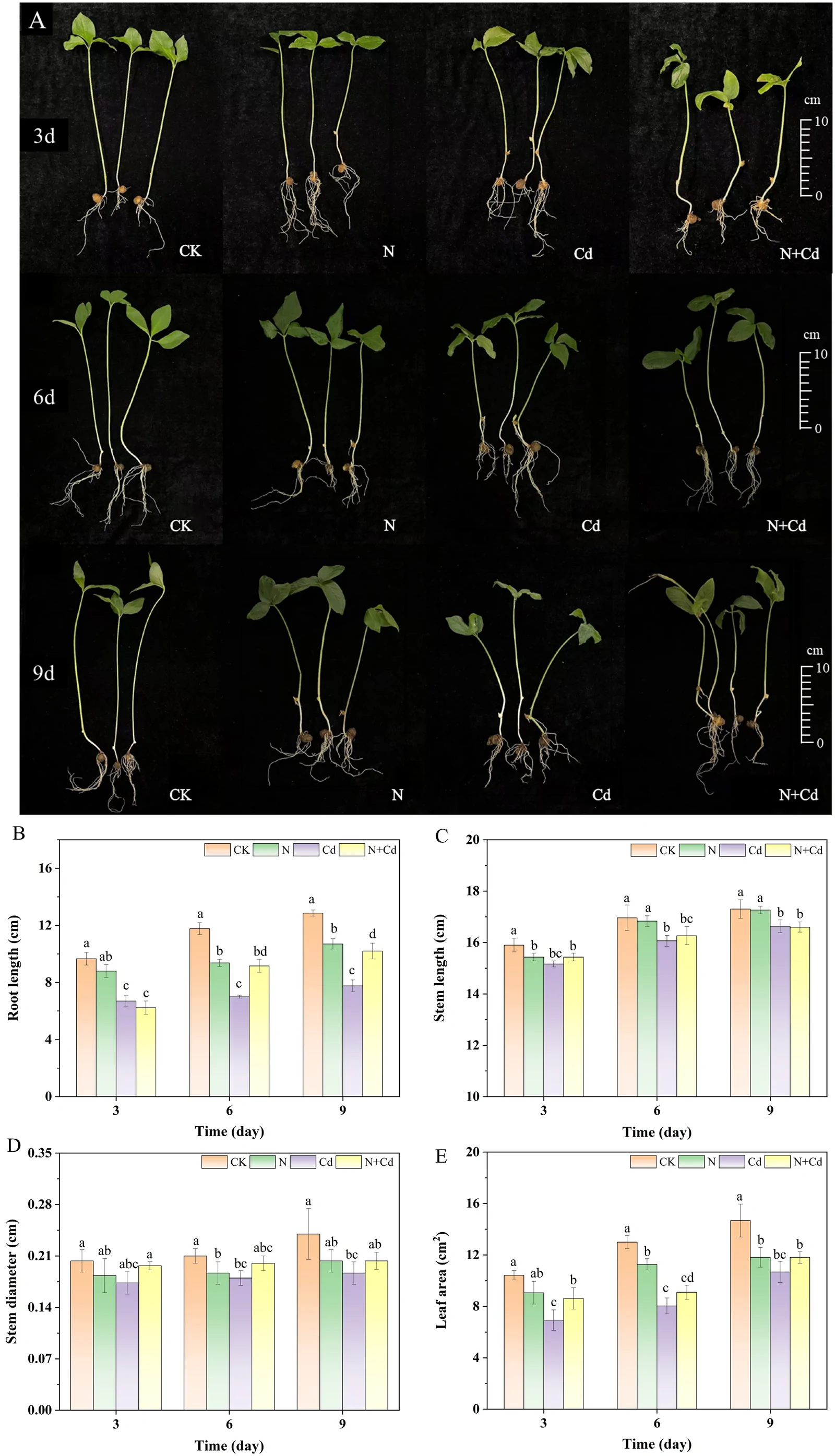
Effects of different treatments on the growth of *P. ternata* seedlings under control (CK), nitrogen (N), cadmium (Cd), and combined nitrogen + cadmium (N+Cd) conditions on days 3, 6, and 9. **(A)** Representative photographs of whole seedlings; the vertical ruler to the right of each row indicates the scale in centimeters (0–10 cm). **(B)** Root length, **(C)** stem length, **(D)** stem diameter, and **(E)** leaf area. The values are the means ± SEs (n = 6). Different lowercase letters above the bars indicate significant differences among treatments at the same time point according to Duncan’s multiple range test (*P* < 0.05).

Compared with that in the CK treatment, root length in the Cd treatment group was significantly reduced at all time points (**Figure 1B**). The root length in the Cd treatment group was significantly lower than that in the CK group at all three time points, with decreases of approximately 30.7%, 40.5%, and 39.6% at days 3, 6, and 9, respectively. The leaf area also significantly decreased under Cd stress, especially at days 3 and 6 (**Figure 1E**). Stem length and stem diameter were significantly lower in the Cd treatment group than in the CK group on days 6 and 9 (**Figures 1C, D**).

Nitrogen application alone (N treatment) generally maintained a growth performance comparable to or only slightly lower than that of the CK, although significant differences were observed at certain time points (e.g., root length and leaf area on day 6).

Importantly, nitrogen supplementation in the N+Cd treatment group partially alleviated Cd-induced growth inhibition. The root length in the N+Cd treatment group was significantly greater than that in the Cd alone group at days 6 and 9, with increases of approximately 31.0% and 31.3%, respectively (**Figure 1B**). The leaf area also partially recovered in the N+Cd treatment group, although it remained lower than that in the CK group on day 9. The effects of nitrogen supplementation on stem length and stem diameter were relatively weak and varied among the sampling times. Overall, these results indicate that exogenous nitrogen alleviated mainly Cd-induced growth inhibition by promoting root elongation and partially improving leaf expansion, with the alleviatory effects becoming more evident at later stages.

### 3.2 Effects of exogenous nitrogen on Cd concentrations and translocation in *P. ternata*

Cd concentrations in the shoots and roots of *P. ternata* showed distinct temporal responses to nitrogen supplementation under Cd stress (**Figure 2**). In the shoots, Cd exposure increased Cd concentrations compared with the CK treatment, with the increase becoming particularly pronounced on day 9 (**Figure 2A**). Compared with the Cd treatment, the N+Cd treatment showed lower shoot Cd concentrations at all three sampling times. Although the differences between the Cd and N+Cd treatments were not significant on days 3 and 6, shoot Cd concentration was significantly reduced by nitrogen supplementation on day 9.

**Figure 2:**
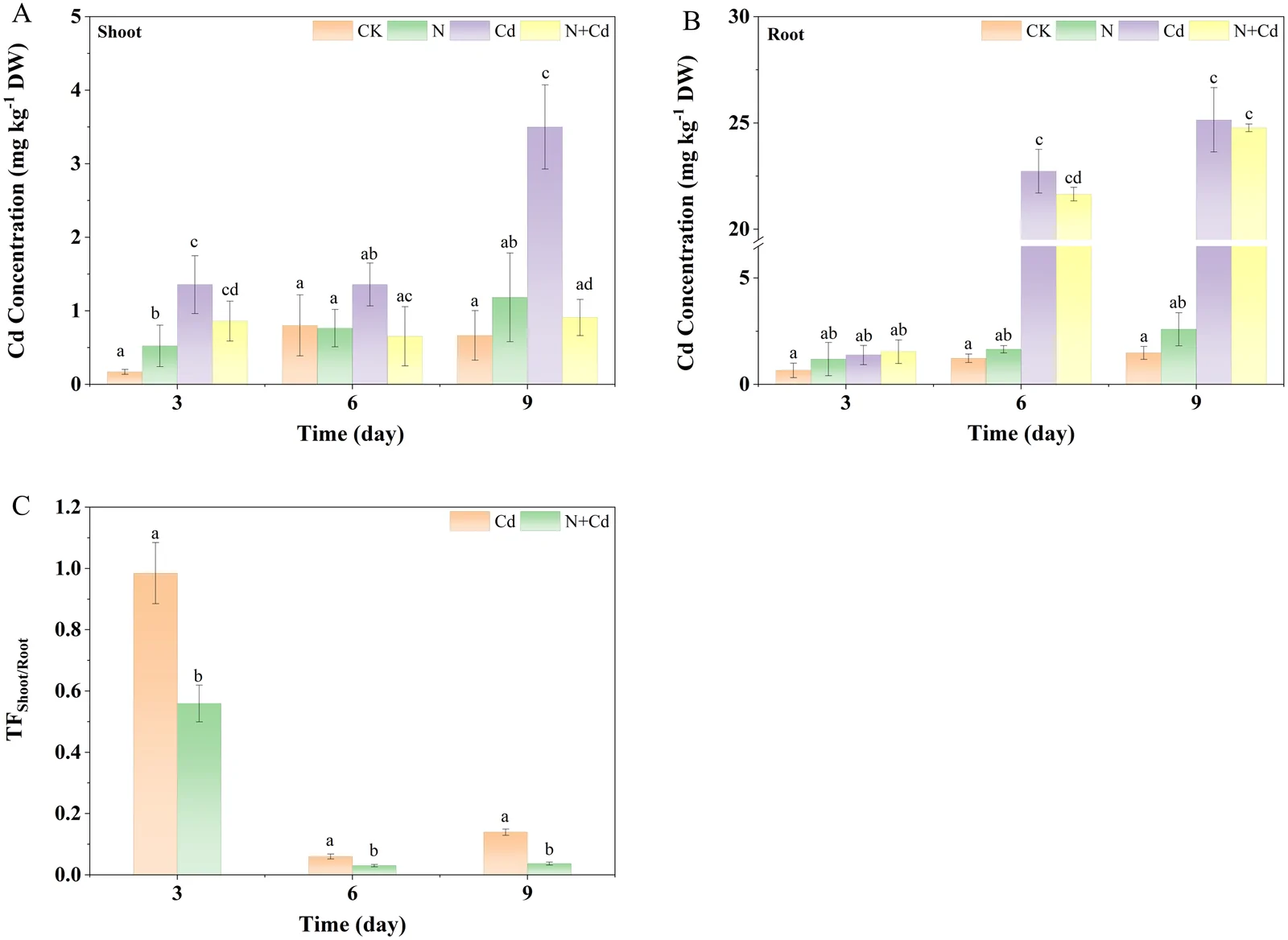
Effects of nitrogen supplementation on Cd concentrations and root-to-shoot translocation in *P. ternata* seedlings. Cd concentrations in the shoots **(A)** and roots **(B)** under control (CK), nitrogen (N), cadmium (Cd), and combined nitrogen + cadmium (N+Cd) treatments on days 3, 6, and 9. **(C)** Root-to-shoot translocation factor (TF) of Cd under the Cd and N+Cd treatments, calculated as the ratio of shoot Cd concentration to root Cd concentration. Values are presented as means ± SEs (n = 6). Different lowercase letters above the bars indicate significant differences among treatments at the same sampling time according to Duncan’s multiple range test (*P* < 0.05).

In the roots, Cd concentrations increased markedly with prolonged Cd exposure, particularly on days 6 and 9 (**Figure 2B**). In contrast to the response observed in the shoots, no significant differences in root Cd concentration were detected between the Cd and N+Cd treatments at any of the three sampling times. These results indicate that nitrogen supplementation had a limited effect on root Cd concentration while reducing shoot Cd concentration at the later stage.

To further assess the translocation of Cd within the plants, the root-to-shoot translocation factor (TF) was calculated as the ratio of shoot Cd concentration to root Cd concentration (**Figure 2C**). Nitrogen supplementation significantly decreased the TF compared with the Cd treatment at all three sampling times (*P* < 0.05), and the reduction was particularly pronounced at the later stages. Taken together, these results indicate that nitrogen supplementation restricted Cd translocation from roots to shoots without markedly altering root Cd concentration, thereby changing Cd partitioning within *P. ternata* under Cd stress.

### 3.3 Effects of exogenous nitrogen on antioxidant enzyme activity and lipid peroxidation under Cd stress

As shown in **Figure 3** and **Supplementary Table 2**, Cd stress affected the antioxidant defense system and membrane lipid peroxidation in *P. ternata*, but the responses varied markedly among enzymes and sampling times.

**Figure 3:**
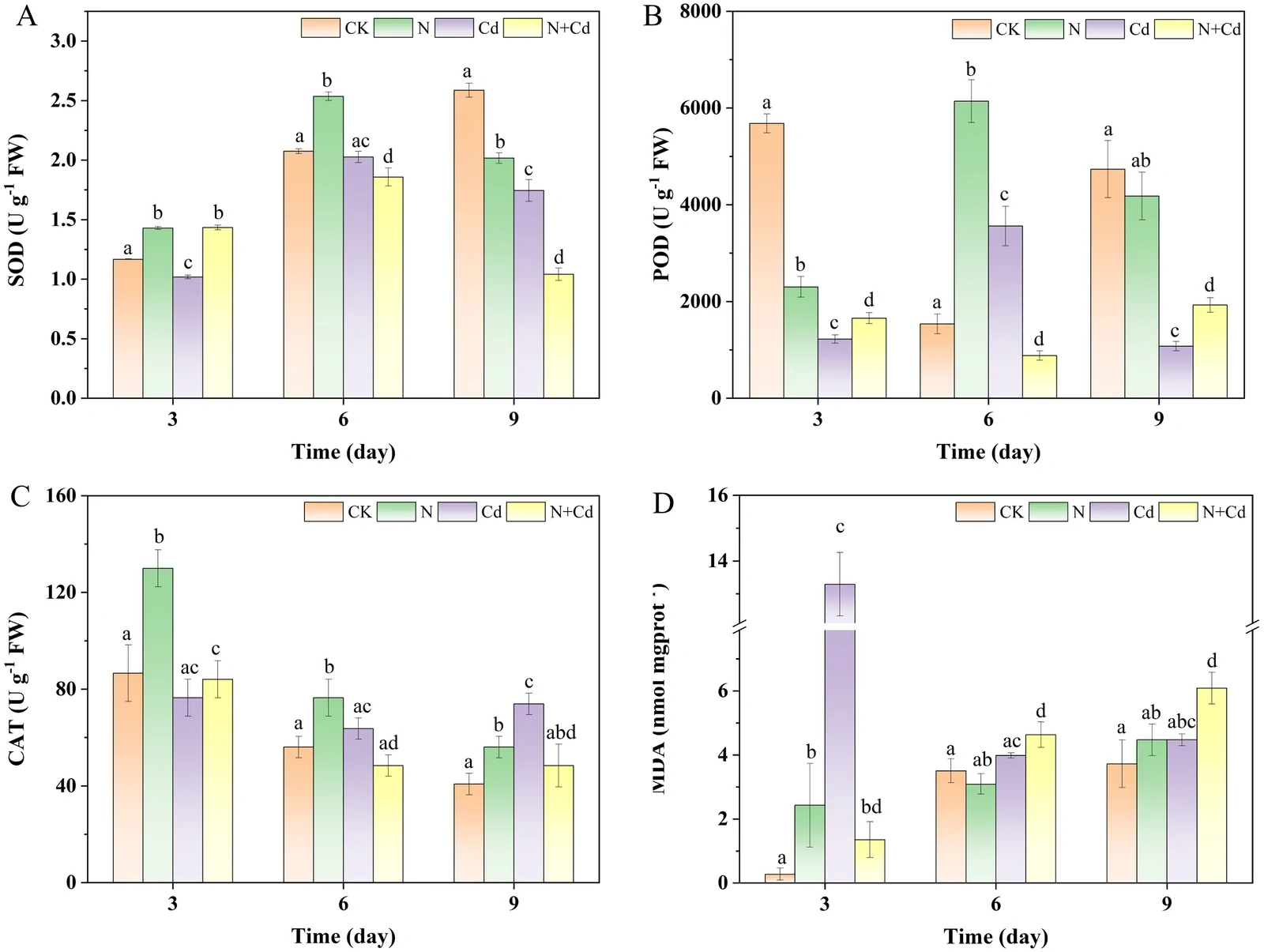
Effects of different treatments on **(A)** SOD activity, **(B)** POD activity, **(C)** CAT activity, and **(D)** MDA content in *P. ternata* seedlings under the CK, N, Cd, and N+Cd treatments on days 3, 6, and 9. The values are the means ± SEs (n = 6). Different lowercase letters above the bars indicate significant differences among treatments at the same time point according to Duncan’s multiple range test (*P* < 0.05).

In terms of SOD activity (**Figure 3A**), compared with the control treatment, the Cd treatment reduced SOD activity at days 3 and 9, whereas the difference between the Cd treatment and control treatment was relatively small on day 6. Nitrogen supplementation alone significantly increased SOD activity at days 3 and 6. In the N+Cd treatment group, SOD activity was restored to a level comparable to that in the N treatment group on day 3, but it decreased at days 6 and 9 and was significantly lower than that in the other treatments on day 9. These results indicate that the effect of nitrogen supplementation on SOD activity under Cd stress strongly depended on time.

The response of POD activity (**Figure 3B**) was more complex. On day 3, compared with the CK treatment, the Cd treatment markedly reduced POD activity, and the POD activity in the N+Cd treatment remained much lower. On day 6, however, compared with that in the CK treatment, POD activity in the Cd treatment was greater, whereas among all the treatments, the N+Cd treatment resulted in the lowest POD activity. By day 9, POD activity in the Cd treatment remained markedly lower than that in the CK, whereas nitrogen supplementation partially restored POD activity in the N+Cd treatment group, although it remained lower than in the CK and N treatments. These results suggest that nitrogen supplementation modulated POD activity under Cd stress, but the direction and magnitude of the response differed across time points.

CAT activity (**Figure 3C**) also exhibited variable responses. Compared with the control treatment, nitrogen application alone consistently increased the CAT activity at all three time points. In contrast, the response of CAT activity to Cd stress was not consistently inhibitory: compared with the control plants, the Cd-treated plants presented slightly lower or comparable CAT activity on day 3, a moderate increase on day 6, and a marked increase on day 9. In the N+Cd treatment group, the CAT activity was close to that in the Cd alone group on day 3, was numerically lower than that in the Cd alone group on day 6 and was significantly lower on day 9. These findings indicate that nitrogen supplementation altered CAT activity under Cd stress, particularly at the later stage.

The MDA content (**Figure 3D**), which serves as an indirect proxy for the extent of membrane lipid peroxidation, increased markedly under Cd stress on day 3 compared with that in the CK. Nitrogen supplementation strongly reduced MDA accumulation in the N+Cd treatment group on day 3, suggesting that nitrogen supplementation has an early protective effect against Cd-induced lipid peroxidation. However, this effect was not sustained at later stages. On day 6, the MDA content in the N+Cd treatment group was greater than that in the Cd alone group, and on day 9, the N+Cd treatment group presented the highest MDA content among all the treatments. These results indicate that the mitigation of membrane lipid peroxidation by nitrogen supplementation was evident mainly at the early stage.

Overall, exogenous nitrogen modulated antioxidant enzyme activities and lipid peroxidation in *P. ternata* under Cd stress. Its protective effect was most evident at the early stage, especially in the restoration of SOD activity and the reduction in MDA accumulation on day 3. However, the responses of POD, CAT, and MDA became more complex at later stages, indicating that nitrogen-mediated regulation of oxidative stress is time dependent rather than uniformly protective throughout the treatment period.

### 3.4 Transcriptomic responses to nitrogen supplementation under Cd stress

To investigate the molecular mechanisms underlying nitrogen-mediated alleviation of Cd toxicity, transcriptome sequencing was performed on *P. ternata* root-tip tissues on days 3, 6, and 9. Differential expression analysis between the N+Cd and Cd treatments was conducted using DESeq2 with the criteria |log_2_FC| > 1 and FDR < 0.05 (Guo et al., 2022).

As shown in the volcano plots (**Figure 4**), nitrogen supplementation induced extensive transcriptomic reprogramming under Cd stress. On day 3 (**Figure 4A**), 36,036 genes were significantly differentially expressed in the N+Cd treatment group compared with those in the Cd alone group, including 17,834 upregulated genes and 18,202 downregulated genes. On day 6 (**Figure 4B**), 9,373 DEGs were detected, with 3,315 upregulated and 6,058 downregulated genes. On day 9 (**Figure 4C**), 33,136 DEGs were identified, including 16,167 upregulated genes and 16,969 downregulated genes.

**Figure 4:**
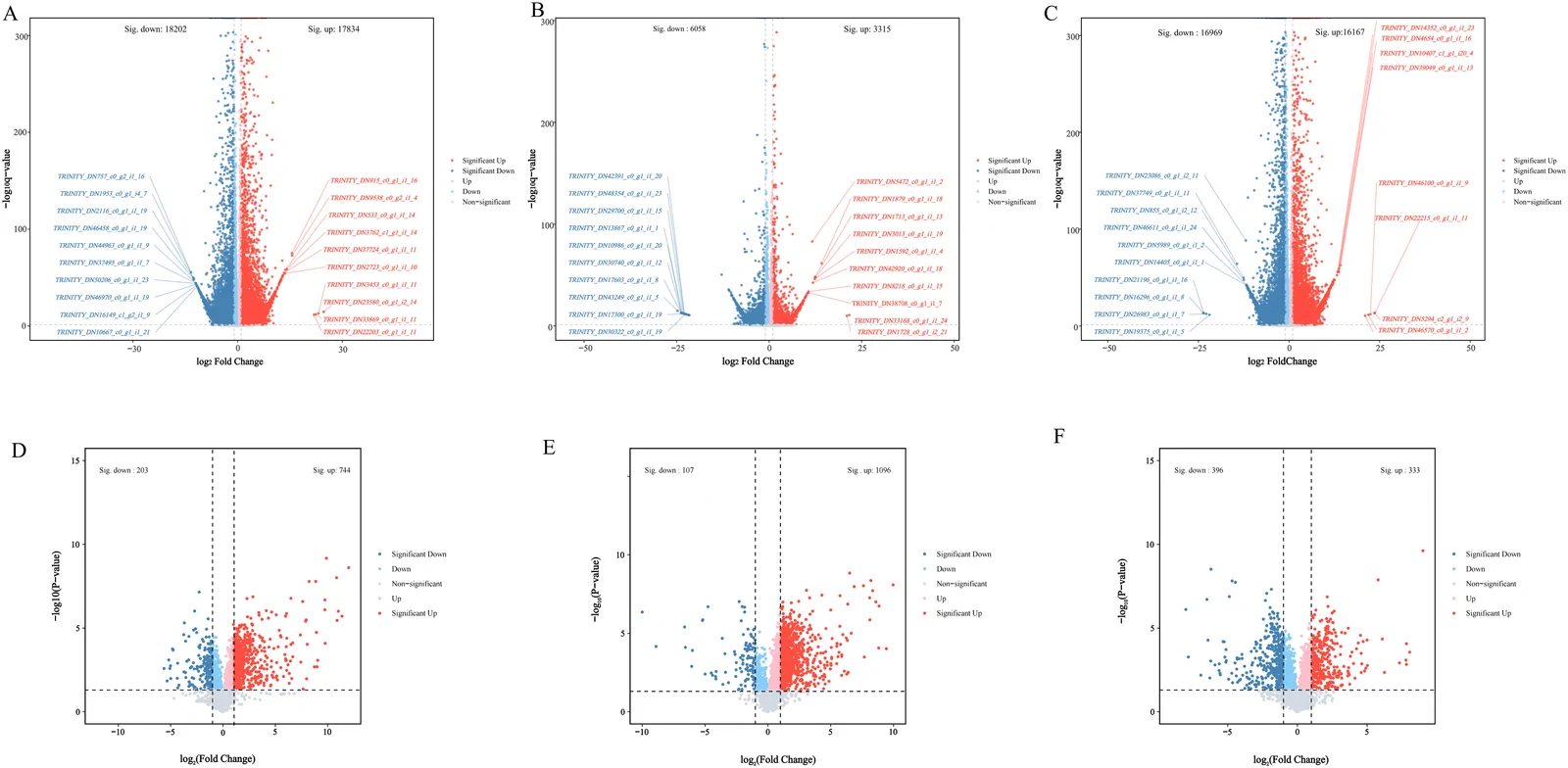
Volcano plots of differentially expressed genes (DEGs) and differentially accumulated metabolites (DAMs) in *P. ternata* roots between the N+Cd and Cd treatments. **(A–C)** DEGs on day 3 **(A)**, day 6 **(B)**, and day 9 **(C)**. Significantly differentially expressed genes were identified using |log_2_FC| > 1 and a false discovery rate (FDR) < 0.05, and representative highly responsive transcripts are labeled in each plot. **(D–F)** DAMs on day 3 **(D)**, day 6 **(E)**, and day 9 **(F)**, identified using VIP > 1.0, |log_2_FC| > 1.0 and *P* < 0.05. In all panels, red and blue dots represent significantly up- and downregulated features, respectively, and gray dots indicate nonsignificant features; the vertical dashed lines indicate the fold-change threshold (|log_2_FC| > 1) and, in **(D–F)**, the horizontal dashed line indicates the significance threshold (*P* < 0.05). “Sig. up” and “Sig. down” denote the numbers of significantly up- and downregulated features in each panel.

The number of DEGs was greatest at days 3 and 9, indicating that nitrogen supplementation triggered strong transcriptional responses during both the early and late stages of Cd stress. In contrast, the number of DEGs was much lower on day 6, suggesting a comparatively moderate transcriptional adjustment at this stage. Several highly responsive transcripts are labeled in the volcano plots, and their gene IDs, NR annotations, regulation patterns, log_2_FC values, and q-values are provided in **Supplementary Tables 3–S5**. Because many of these transcripts were annotated as hypothetical proteins or lacked clear functional annotation, their biological roles require further validation.

Overall, these transcriptomic results indicate that exogenous nitrogen markedly altered gene expression patterns in *P. ternata* roots under Cd stress. These dynamic transcriptional changes provided a basis for subsequent pathway-level analyses, particularly those related to amino acid metabolism, antioxidant responses, detoxification-related processes, and arginine and proline metabolism.

Although ko00330 (arginine and proline metabolism) did not reach statistical significance in the transcriptomic KEGG enrichment analysis, 42, 20, and 29 DEGs were annotated to this pathway in the N+Cd vs CK comparison on days 3, 6, and 9, respectively.

### 3.5 Metabolic reprogramming in roots under Cd stress with nitrogen supplementation

Untargeted metabolomic profiling was conducted on root tissues to investigate exogenous nitrogen-mediated metabolic reprogramming under Cd stress. Differentially accumulated metabolites (DAMs) between the N+Cd and Cd treatments were identified using VIP > 1.0, |log_2_(fold change)| > 1.0, and *P* < 0.05 (Duan et al., 2025).

As shown in the volcano plots (**Figures 4D–F**), nitrogen supplementation induced extensive and dynamic metabolic changes under Cd stress. On day 3 (**Figure 4D**), 947 metabolites were significantly altered (744 upregulated and 203 downregulated) in the N+Cd treatment group compared with those in the Cd alone group. The strongest metabolic response occurred on day 6 (**Figure 4E**), with 1,203 metabolites significantly altered (1,096 upregulated and 107 downregulated). On day 9 (**Figure 4F**), 729 metabolites were significantly different (333 upregulated and 396 downregulated).

Further examination of representative differentially accumulated metabolites revealed that nitrogen supplementation induced time-dependent metabolic changes in *P. ternata* roots under Cd stress (**Figure 5**). On day 3, nitrogen supplementation affected mainly carbohydrates and related primary metabolites. Several metabolites, including sucrose, β-D-galactose, galactonic acid, myo-inositol, and N-acetylisoputreanine, were downregulated, whereas cystinylglycine, pyruvylglycine, O-glutarylcarnitine, hyaluronic acid, and 4′-azidocytidine were upregulated (**Figure 5A**). On day 6, the metabolic response shifted toward sulfated, conjugated, and secondary metabolites. Compounds such as 3-sulfopyruvic acid, genistein sulfate, dopamine 4-sulfate, dUDP, and luteolin-related derivatives were downregulated, whereas N²-γ-glutamylglutamine, erioposide A, 3′-sialyllactose, 4′-azidocytidine, and several vitamin D-related derivatives were upregulated (**Figure 5B**). On day 9, nitrogen supplementation continued to reshape secondary metabolism and amino acid-related metabolism. Some secondary metabolites, including nagilactone F, albafuran A, prosafrinine, D-methionine (S)-S-oxide, and 4-O-caffeoylshikimic acid, were downregulated, whereas the levels of dendrobine, trigonelline, N²-γ-glutamylglutamine, L-glutamine, and salicylamide glucuronide were upregulated (**Figure 5C**). Overall, nitrogen supplementation did not cause a uniform increase in all metabolite classes. Instead, it reshaped both primary and secondary metabolism in a time-dependent manner, with nitrogen- and amino acid-related metabolites, particularly N²-γ-glutamylglutamine and L-glutamine, showing notable accumulation at the middle and later stages.

**Figure 5:**
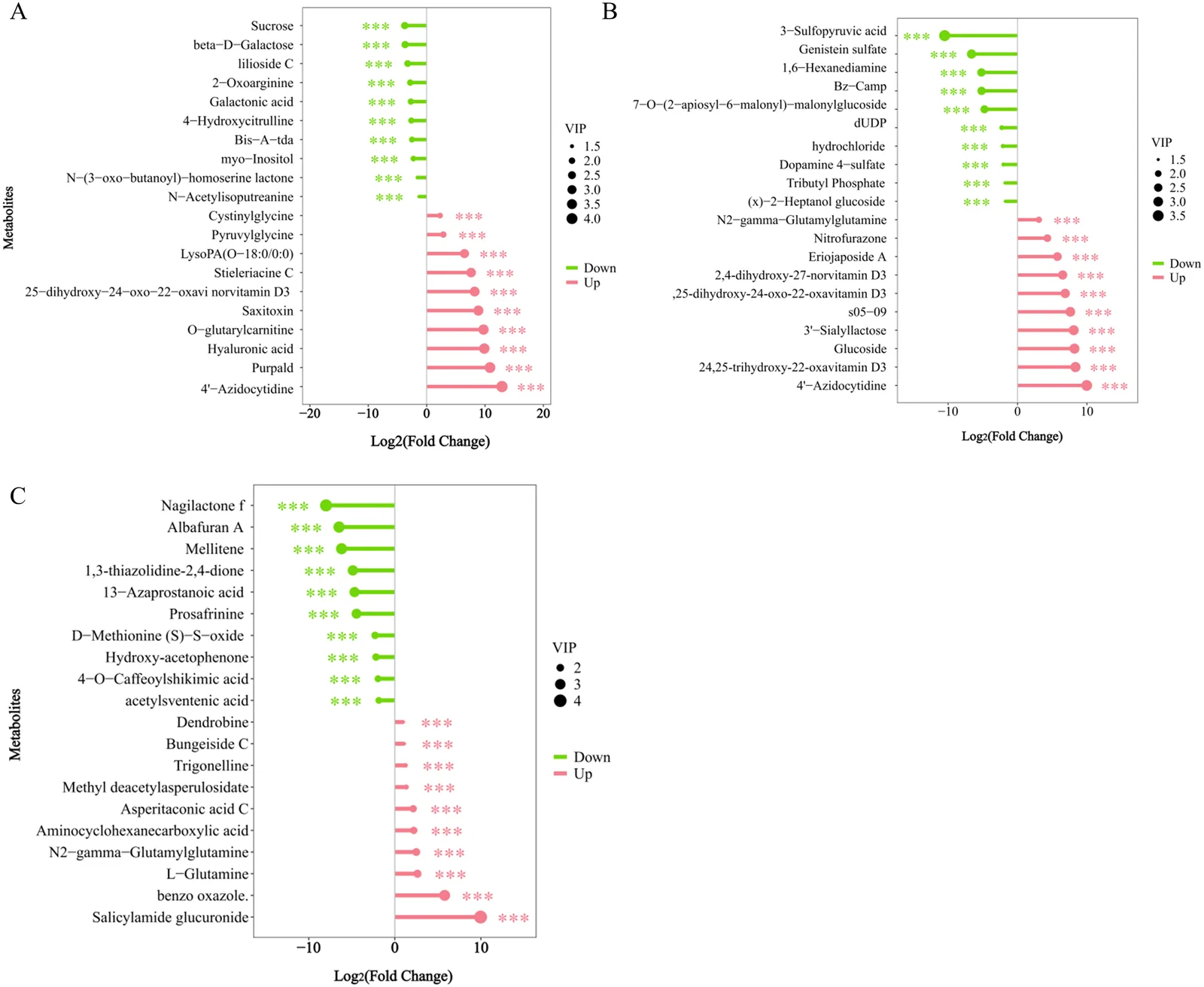
Representative differentially accumulated metabolites in *P. ternata* roots under the N+Cd treatment compared with those under the Cd treatment on day 3 **(A)**, day 6 **(B)**, and day 9 **(C)**. The green and pink dots indicate downregulated and upregulated metabolites, respectively. The circle size represents the VIP value. Asterisks (***) indicate significant differences at *P* < 0.001, respectively.

### 3.6 Venn diagram and KEGG pathway enrichment analysis of differentially accumulated metabolites

Venn diagram analysis (**Figure 6**) was performed separately for each sampling time point to compare the DAMs among the Cd, N, and N+Cd treatments. For each time point, the DAMs were defined by comparing each treatment with the CK before overlap analysis (Al-Khayri et al., 2023). On day 3 (**Figure 6A**), 686 metabolites (31.9%) were commonly regulated across all three treatments, while the N+Cd treatment resulted in the production of 189 unique metabolites (8.8%). The overlap between N+Cd and N (345 metabolites, 16.0%) was substantially greater than that between N+Cd and Cd (138 metabolites, 6.4%). On day 6 (**Figure 6B**), the three-way common set decreased to 578 metabolites (27.9%), and the N+Cd treatment resulted in a greater number of unique metabolites (306, 14.8%). The overlap between N+Cd and N remained prominent (460 metabolites, 22.2%), whereas the overlap between N+Cd and Cd was relatively small (139 metabolites, 6.7%). By day 9 (**Figure 6C**), the three-way overlap increased markedly to 864 metabolites (40.9%), and the number of unique metabolites in the N+Cd treatment decreased to 166 (7.9%). At this later stage, the N+Cd-Cd overlap (256 metabolites, 12.1%) exceeded the N+Cd-N overlap (191 metabolites, 9.0%), indicating a partial shift in metabolic similarity over time. A comprehensive petal diagram integrating all nine treatment–time combinations (**Figure 6D**) revealed a persistent core of 182 metabolites that were commonly accumulated across all the treatments and sampling times.

**Figure 6:**
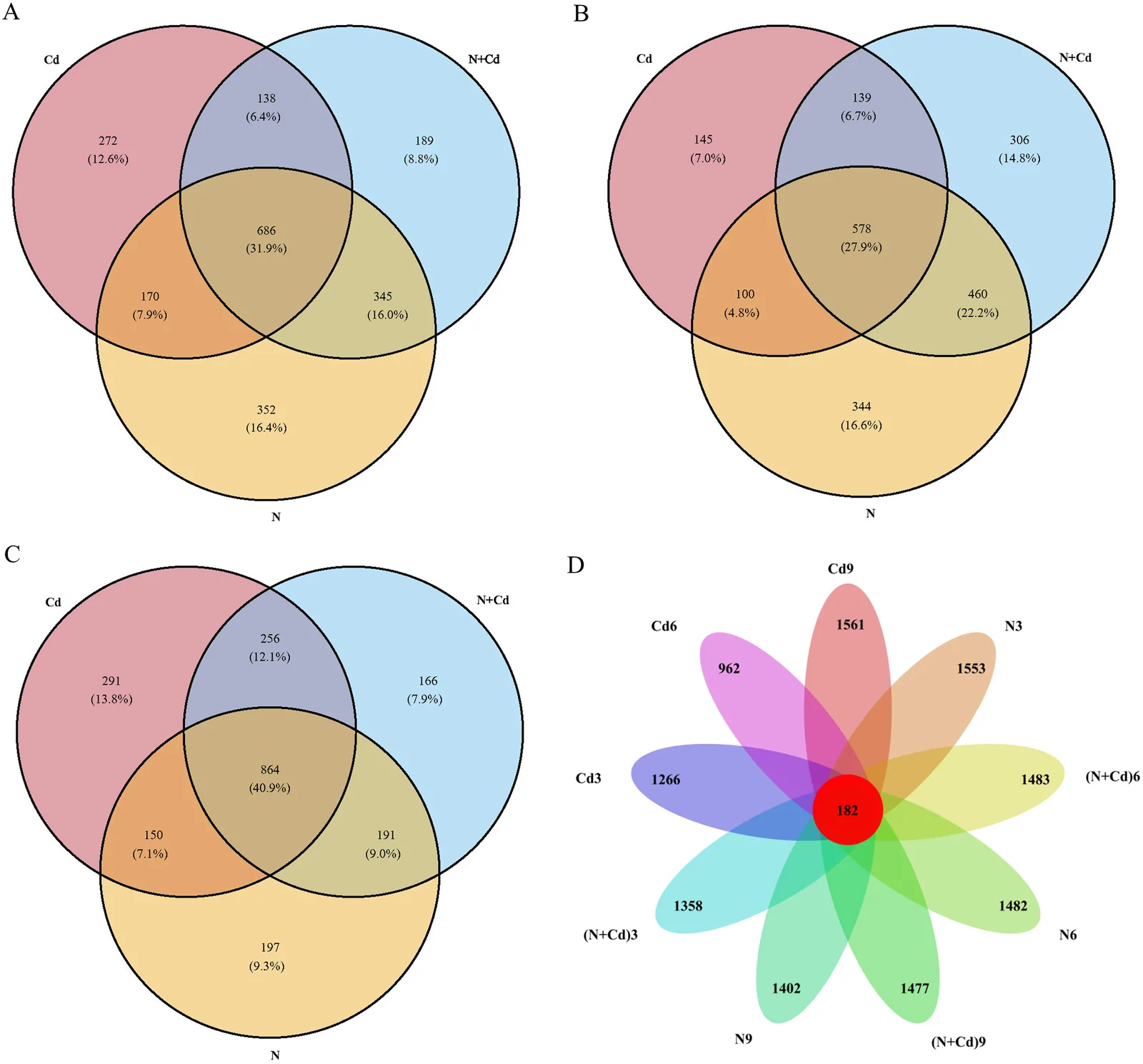
Venn diagrams showing the overlap of DAMs in *P. ternata* roots. **(A–C)** Three-way Venn diagrams of DAMs among the Cd, N and N+Cd treatments on day 3 **(A)**, day 6 **(B)**, and day 9 **(C)**. **(D)** Petal diagram showing the number of shared and unique DAMs across all treatment groups and sampling times. The numbers represent metabolite counts, with percentages shown in parentheses.

KEGG pathway enrichment analysis (**Figure 7**) further showed that these DAMs were enriched in multiple stress-related pathways. On day 3 (**Figure 7A**), ascorbate and aldarate metabolism, ABC transporters, galactose metabolism, purine metabolism, and arginine and proline metabolism were among the most prominently enriched pathways. On day 6 (**Figure 7B**), amino acid metabolism pathways, including alanine, aspartate and glutamine metabolism; histidine metabolism; arginine biosynthesis; and arginine and proline metabolism, were highly enriched, as were pentose and glucuronate interconversions. On day 9 (**Figure 7C**), histidine metabolism, pyrimidine metabolism, arginine and proline metabolism, alanine, aspartate and glutamine metabolism, purine metabolism, and nitrogen metabolism remained among the top enriched pathways. Notably, arginine and proline metabolism were consistently enriched across all three time points, suggesting its potential importance in nitrogen-driven metabolic reprogramming under Cd stress. In addition, multiple comparisons revealed that glutathione metabolism and ABC transporters were enriched. These results highlight the importance of amino acid metabolism, particularly arginine and proline metabolism, in the exogenous nitrogen-mediated alleviation of Cd toxicity.

**Figure 7:**
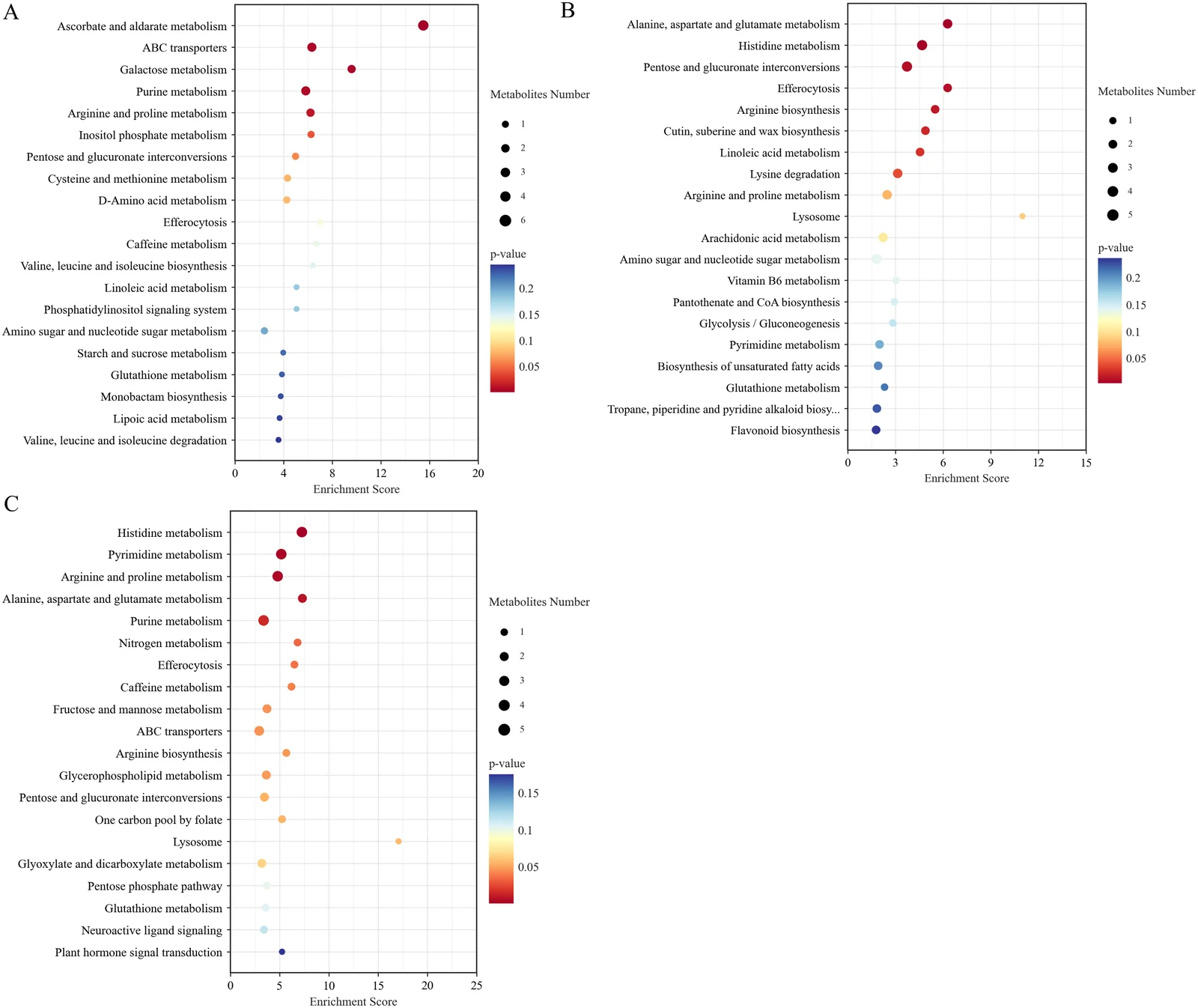
KEGG pathway enrichment analysis of differentially accumulated metabolites between the N+Cd and Cd treatments in *P. ternata* roots on day 3 **(A)**, day 6 **(B)**, and day 9 **(C)**. The enrichment score is shown on the x-axis. Bubble color represents the P value, and bubble size indicates the number of enriched metabolites.

### 3.7 Effects of nitrogen supplementation on rhizosphere microbial composition and richness under Cd stress

To further investigate rhizosphere microbial responses to nitrogen supplementation under Cd stress (Ng et al., 2023), Circos plots were constructed, and alpha diversity analyses were performed on days 3, 6, and 9 (**Figures 8A–C**). The rhizosphere microbial community was represented mainly by *Proteobacteria*, *Bacteroidota*, and *Firmicutes*. Cd stress altered the associations between samples and several dominant ASVs, indicating a shift in rhizosphere microbial composition under Cd exposure. Compared with Cd alone, the N+Cd treatment partially maintained the distribution of dominant ASVs at later sampling stages. Alpha diversity analysis (**Figure 8D**) revealed that the ACE richness index was broadly maintained in the N+Cd treatment under Cd stress, although the magnitude of the nitrogen effect varied across time points. These results suggest that exogenous nitrogen may help reduce Cd-induced disturbance of the rhizosphere microbial community, although the functional significance and causal contribution of these microbial shifts require further experimental validation.

**Figure 8:**
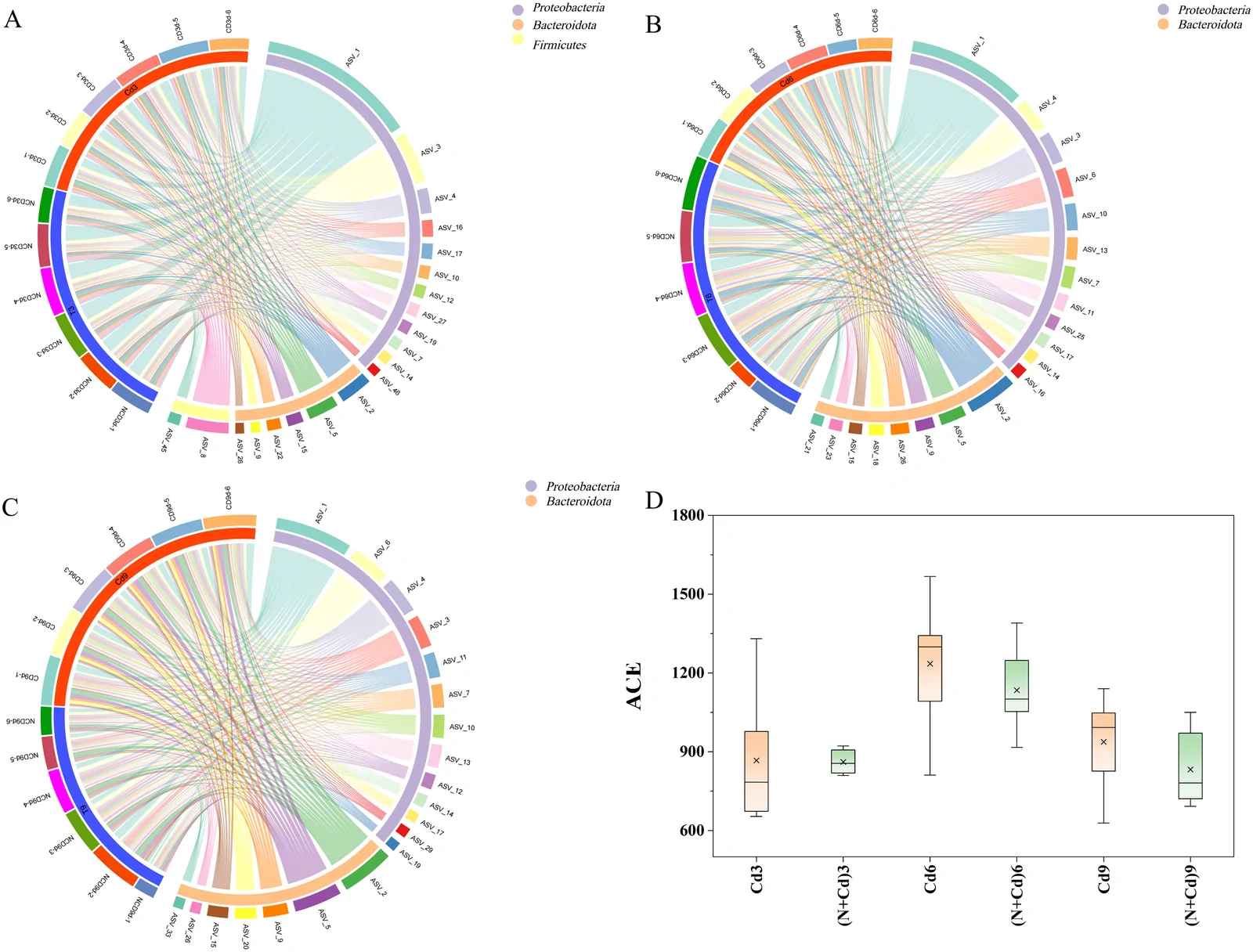
Effects of nitrogen supplementation on the rhizosphere microbial community of *P. ternata* under Cd stress. **(A–C)** Circos plots showing the relationships between samples and dominant ASVs on day 3 **(A)**, day 6 **(B)**, and day 9 **(C)**. The left semicircle represents samples, and the right semicircle represents dominant ASVs, with ribbons indicating relative abundance connections. ASVs are colored by phylum. **(D)** Boxplot of the ACE richness index in the Cd and N+Cd treatments at different time points.

Beta diversity analysis based on NMDS ordination revealed a distinct separation in the rhizosphere bacterial community structure between the N+Cd and Cd treatments at all three sampling time points (stress = 0.008–0.014; **Supplementary Figure 1**). PICRUSt2-based functional prediction at 6 days post-treatment revealed several significantly altered COG functional categories between treatments (**Supplementary Figure 2**), including COG1012, COG0841, and COG0491.

### 3.8 Expression profiles of arginine and proline metabolism-related genes

To further elucidate the molecular mechanisms underlying nitrogen-mediated alleviation of Cd stress, the expression profiles of DEGs and the relative abundances of metabolites associated with arginine and proline metabolism were analyzed on days 3, 6, and 9 (**Figure 9**; **Supplementary Table 6**).

**Figure 9:**
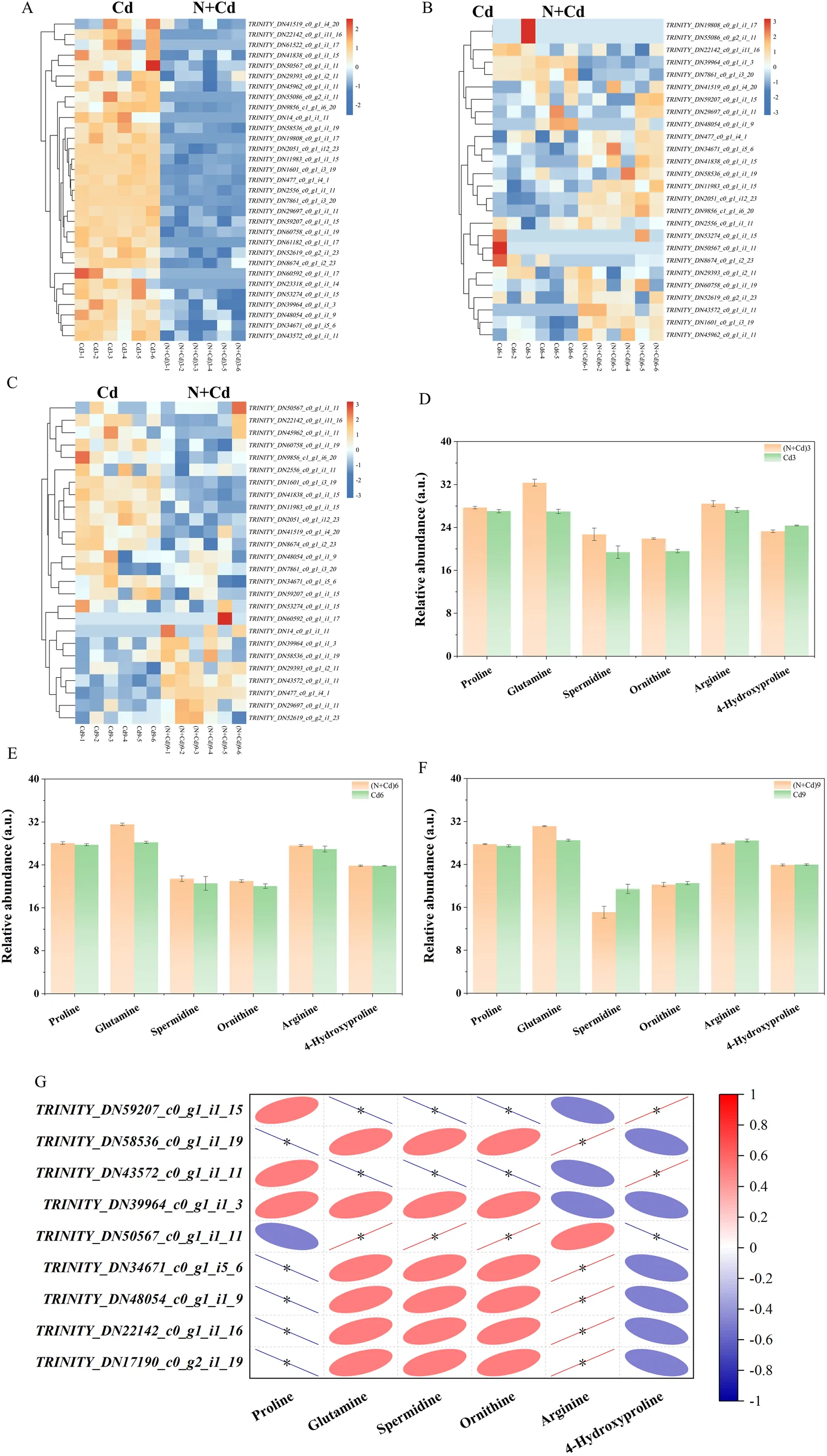
Integrated transcriptomic and metabolomic profiles of DEGs and metabolites involved in arginine and proline metabolism in *P. ternata* roots under Cd and N+Cd treatments. **(A–C)** Hierarchical clustering heatmaps showing the expression profiles of DEGs involved in arginine and proline metabolism on day 3 **(A)**, day 6 **(B)**, and day 9 **(C)**. The color scale represents Z score-normalized expression values (red: relatively high expression; blue: relatively low expression). **(D–F)** Relative abundances (normalized peak areas) of key metabolites annotated to the arginine and proline metabolism pathway (ko00330) on day 3 **(D)**, day 6 **(E)**, and day 9 **(F)**. **(G)** Spearman correlation analysis between DEGs and key metabolites in the arginine and proline metabolism pathway. The circle size and color intensity represent the magnitude of the Spearman correlation coefficient (r); red indicates a positive correlation, and blue indicates a negative correlation. Asterisks indicate statistically significant correlations (*P < 0.05).

As shown in the hierarchical clustering heatmaps (**Figures 9A–C**), nitrogen supplementation markedly altered the expression profiles of genes in this pathway under Cd stress. Several key genes with functional annotations were upregulated in the N+Cd treatment group compared with those in the Cd alone group. These genes included *TRINITY_DN59207_c0_g1_i1_15*, encoding pyrroline-5-carboxylate reductase (P5CR), a rate-limiting enzyme in proline biosynthesis; *TRINITY_DN43572_c0_g1_i1_11*, encoding 1-pyrroline-5-carboxylate dehydrogenase; *TRINITY_DN50567_c0_g1_i1_11*, encoding a spermidine resistance protein; *TRINITY_DN58536_c0_g1_i1_19*, encoding spermidine synthase; and multiple aldehyde dehydrogenase genes, such as *TRINITY_DN39964_c0_g1_i1_3*, encoding aldehyde dehydrogenase 2B2. On day 3 (**Figure 9A**), the expression of most of these genes increased in the N+Cd treatment group. The differential expression pattern became more pronounced on day 6 (**Figure 9B**), with a distinct cluster of genes strongly upregulated in the N+Cd group. By day 9 (**Figure 9C**), several key genes maintained higher transcript abundance, suggesting sustained activation of arginine and proline metabolism during nitrogen-mediated Cd tolerance.

Consistent with these transcriptional changes, metabolite profiling revealed corresponding alterations in key metabolites within this pathway (**Figures 9D–F**). The relative abundances of proline were greater in the N+Cd treatment group than in the Cd alone group across all three time points, which is consistent with the upregulation of P5CR and 1-pyrroline-5-carboxylate dehydrogenase. Similarly, glutamine and spermidine abundances increased under N+Cd treatment, particularly on day 6 and day 9, mirroring the transcriptional activation of spermidine synthase. These metabolic changes suggest increased flux through the arginine and proline metabolism pathways in response to nitrogen supplementation.

To further elucidate coordinated regulatory patterns across the transcriptomic and metabolomic layers, as revealed by Spearman correlation analysis (**Figure 9G**), *TRINITY_DN59207_c0_g1_i1_15* (P5CR) was shown to be significantly positively correlated with glutamine and arginine abundances (*P* < 0.05), while *TRINITY_DN43572_c0_g1_i1_11* exhibited significant positive correlations with glutamine and spermidine. Notably, *TRINITY_DN58536_c0_g1_i1_19* (spermidine synthase) was significantly negatively correlated with spermidine abundances, suggesting rapid metabolic turnover of spermidine under N+Cd treatment. Overall, these results indicate concurrent changes at the transcript and metabolite levels within the arginine and proline metabolism pathway, although the directions of the correlations were not uniform.

## 4 Discussion

### 4.1 Nitrogen supplementation alleviates Cd-induced growth inhibition and modulates Cd translocation and oxidative responses

In the present study, exogenous nitrogen supplementation (150 mg N kg^-^¹ supplied as urea) alleviated Cd-induced growth inhibition in *P. ternata* seedlings. Cd stress markedly suppressed plant growth, as reflected by reductions in root length, leaf area, stem length, and stem diameter (**Figure 1**; **Supplementary Table 1**), consistent with the well-documented inhibitory effects of Cd on plant development (Fan et al., 2023; Hu et al., 2025; Yu et al., 2025). Compared with the Cd treatment, the N+Cd treatment significantly increased root length by approximately 31% on days 6 and 9 and partially improved leaf area by day 9. Nitrogen application alone generally maintained plant growth within a moderate range, although some parameters differed from those of the CK at specific time points. These observations are consistent with previous reports showing that an adequate nitrogen supply can improve plant performance under Cd stress by supporting root development, nutrient metabolism, and stress-defense processes (Yang et al., 2020; He et al., 2021; Zhang et al., 2024).

Cd distribution analysis further demonstrated that nitrogen supplementation altered Cd partitioning within *P. ternata*. Root Cd concentrations did not differ significantly between the Cd and N+Cd treatments at any sampling time, whereas the root-to-shoot translocation factor was significantly reduced by nitrogen supplementation on days 3, 6, and 9, accompanied by a significant decrease in shoot Cd concentration on day 9 (**Figure 2**). This pattern suggests that the effect of nitrogen supplementation was associated primarily with restricted root-to-shoot Cd translocation rather than with a marked reduction in root Cd concentration. Similar studies have shown that nitrogen availability can influence Cd uptake, transport, and internal partitioning through changes in plant nutritional status and metal-handling processes (Yang et al., 2020; Hu et al., 2025). However, because the subcellular distribution and chemical speciation of Cd were not determined in the present study, the mechanisms underlying the altered Cd partitioning remain to be established.

Substrate sterilization was expected to initially suppress microbial activity, while soil pH is known to influence nitrification processes, and chloride availability may affect ion uptake and transport in plants. These factors should be considered when interpreting the present short-term responses. Nitrogen supplementation also exerted a time-dependent influence on the antioxidant system and membrane lipid peroxidation. The antioxidant enzymes examined here participate in coordinated ROS metabolism: SOD dismutates superoxide radicals (O_2_·^-^) into hydrogen peroxide (H_2_O_2_), which can subsequently be detoxified by CAT and POD. Under Cd stress, disruption of this antioxidant network can promote ROS accumulation and membrane lipid peroxidation (Balestrasse et al., 2005; Fan et al., 2023; Hu et al., 2025). In the present study, nitrogen supplementation restored SOD activity and markedly reduced MDA accumulation on day 3, indicating an improved early-stage antioxidant response. However, the responses of SOD, POD, CAT, and MDA became more variable at later sampling times, showing that nitrogen-mediated regulation of oxidative stress was not uniformly protective throughout the experiment. Because H_2_O_2_ and O_2_·^-^ were not directly quantified, the interpretation of ROS metabolism is based on antioxidant enzyme activities and MDA, the latter serving only as an indirect proxy for membrane lipid peroxidation. Direct ROS measurements combined with histochemical analyses such as DAB and NBT staining are therefore needed to further clarify these responses.

### 4.2 Nitrogen-mediated Cd tolerance is associated with arginine and proline metabolism and coordinated molecular and rhizosphere responses

Integrated transcriptomic and metabolomic analyses revealed extensive and time-dependent molecular reprogramming in *P. ternata* roots following nitrogen supplementation under Cd stress. KEGG enrichment analysis of the DAMs consistently highlighted arginine and proline metabolism (ko00330) across the three sampling times, indicating that this pathway was closely associated with the metabolic response to nitrogen supplementation. Importantly, however, ko00330 was not significantly enriched in the transcriptomic KEGG analysis in any comparison or at any sampling time (enrichment scores 0.81–1.17, all *P* > 0.05; **Supplementary Tables 7–S9**), although 15–78 transcripts per contrast were annotated to this pathway and several functionally annotated genes showed treatment-dependent expression (**Figures 9A–C**). Therefore, arginine and proline metabolism should be regarded as a pathway identified primarily at the metabolite level rather than as a transcriptionally enriched pathway. The observed transcriptome–metabolome divergence may reflect contributions from post-transcriptional or post-translational regulation, enzyme activity, substrate availability, or other metabolic regulatory processes, none of which were directly examined in the present study.

Arginine and proline metabolism is widely involved in plant responses to environmental stress through osmotic adjustment, ROS homeostasis, protein and membrane stabilization, and polyamine-associated stress signaling (Meena et al., 2019; Spormann et al., 2023). In the present study, nitrogen supplementation altered the transcript abundance of several genes associated with this pathway, including those encoding pyrroline-5-carboxylate reductase (P5CR), 1-pyrroline-5-carboxylate dehydrogenase, spermidine synthase, spermidine resistance protein, and aldehyde dehydrogenase family members (**Figures 9A–C**; **Supplementary Table 6**). These transcriptional changes were accompanied by differences in the relative abundances of proline, glutamine, spermidine, and other pathway-associated metabolites, as estimated from normalized peak areas in the untargeted metabolomic analysis (**Figures 9D–F**). Spearman correlation analysis further revealed significant associations between several transcripts and metabolites: P5CR transcript abundance was positively correlated with the relative abundances of glutamine and arginine, 1-pyrroline-5-carboxylate dehydrogenase was positively correlated with glutamine and spermidine, and spermidine synthase showed a negative correlation with spermidine abundance (**Figure 9G**). The latter represents an inverse association rather than direct evidence of enhanced spermidine turnover. Overall, these concurrent transcriptomic and metabolic changes support an association between arginine and proline metabolism and nitrogen-mediated Cd tolerance. Targeted quantitative metabolite analysis, qRT-PCR validation of key genes, and functional assays are required in future studies.

In addition to arginine and proline metabolism, several other pathways were enriched under nitrogen supplementation, including ascorbate and aldarate metabolism, glutathione metabolism, ABC transporters, alanine, aspartate and glutamine metabolism, histidine metabolism, purine metabolism, and nitrogen metabolism (**Figure 7**). These pathways collectively point to coordinated changes in redox regulation, nitrogen and amino acid metabolism, cellular transport, and stress-response processes (Al-Khayri et al., 2023; Zhang et al., 2024). In particular, glutathione and related thiol compounds are important components of antioxidant defense and metal complexation, whereas ABC transporters participate broadly in cellular transport processes associated with stress responses (Hu et al., 2025). Their enrichment may therefore be consistent with altered cellular Cd handling and the reduced root-to-shoot Cd translocation observed at the physiological level.

Rhizosphere microbial responses provided an additional layer of evidence for nitrogen-associated changes under Cd stress. Circos plots and ACE richness analysis indicated that nitrogen supplementation partly maintained the distribution of dominant ASVs and microbial richness, although the magnitude of the response varied among sampling times (**Figure 8**). NMDS analysis further revealed clear separation between the Cd and N+Cd rhizosphere bacterial communities at all three sampling times (stress = 0.008–0.014; **Supplementary Figure 1**), indicating treatment-associated shifts in community structure. PICRUSt2-based functional prediction identified several differentially represented COG categories on day 6 (**Supplementary Figure 2**), including COG1012 (NAD-dependent aldehyde dehydrogenase), COG0841 (cation/multidrug efflux pump), and COG0491 (glyoxalase II). The occurrence of an aldehyde dehydrogenase-related functional category is noteworthy in view of the host-side changes associated with proline and aldehyde metabolism. However, PICRUSt2 provides predicted functional potential rather than direct measurements of microbial gene expression or activity. Similarly, the enrichment of efflux- and detoxification-related predicted functions cannot establish a direct microbial contribution to Cd tolerance. Nitrogen-driven changes in plant growth, nutrient availability, and root metabolism may themselves influence rhizosphere community assembly (Ng et al., 2023). Therefore, the functional significance of these microbial shifts should be interpreted cautiously and further evaluated through metagenomic or meta transcriptomic approaches, root exudate profiling, and microbiome manipulation experiments. Moreover, a direct integrative correlation analysis linking the rhizosphere microbiome with the transcriptomic and metabolomic datasets was not performed in the present study and represents an important direction for future work.

Taken together, these findings suggest that nitrogen supplementation is associated with improved Cd tolerance in *P. ternata* through coordinated changes involving restricted root-to-shoot Cd translocation, arginine and proline metabolism, antioxidant and transport-related processes, and rhizosphere microbial responses. Rather than acting through a single pathway, nitrogen supplementation appears to influence multiple physiological, molecular, metabolic, and rhizosphere-associated components that together are associated with the alleviation of Cd-induced stress. This interpretation is consistent with previous studies showing that nitrogen availability can influence antioxidant defense, amino acid metabolism, Cd transport, and plant–microbe interactions under heavy-metal stress (Yang et al., 2020; Al-Khayri et al., 2023; Zhang et al., 2024). A proposed framework summarizing these coordinated responses is presented in **Supplementary Figure 3**.

## 5 Conclusions

This study showed that exogenous nitrogen supplementation (150 mg N kg^-^¹ supplied as urea) alleviated Cd-induced stress in *P. ternata* seedlings. Nitrogen application improved plant growth, most notably root length, which increased by approximately 31% on days 6 and 9, and modulated antioxidant enzyme responses while reducing early-stage membrane lipid peroxidation, although these protective effects were time dependent rather than uniformly sustained. Importantly, nitrogen supplementation significantly reduced the root-to-shoot Cd translocation factor at all three sampling times and decreased shoot Cd concentration at the later stage, while root Cd concentrations remained largely unchanged. These findings indicate that nitrogen supplementation primarily altered Cd partitioning within the plant by restricting its translocation from roots to shoots rather than markedly reducing root Cd concentration.

Integrated transcriptomic and metabolomic analyses further revealed extensive molecular and metabolic reprogramming under nitrogen supplementation. Arginine and proline metabolism (ko00330) was enriched among the differentially accumulated metabolites across all three sampling times and was therefore identified as a pathway of particular interest, although its enrichment in the transcriptomic KEGG analysis did not reach statistical significance. Within this pathway, genes encoding pyrroline-5-carboxylate reductase, 1-pyrroline-5-carboxylate dehydrogenase, spermidine synthase, and aldehyde dehydrogenase family members showed treatment-dependent differential expression, accompanied by concurrent changes in the relative abundances of proline, glutamine, and polyamines. Auxiliary pathways, including ascorbate and aldarate metabolism, glutathione metabolism, ABC transporters, and nitrogen metabolism, were also enriched, consistent with coordinated changes in antioxidant defense, cellular transport, and stress-response processes. In parallel, nitrogen supplementation partially maintained rhizosphere microbial structure and richness, while NMDS ordination and PICRUSt2-based prediction revealed treatment-associated shifts in microbial community structure and predicted functional categories.

## Data Availability

The raw sequencing data generated in this study have been deposited in the NCBI Sequence Read Archive (SRA) under BioProject accession number PRJNA1528944.

## References

[B1] Al-Khayri J. M. Banadka A. Rashmi R. Nagella P. Alessa F. M. Almaghasla M. I. . (2023). Cadmium toxicity in medicinal plants: an overview of the tolerance strategies, biotechnological and omics approaches to alleviate metal stress. Front. Plant Sci. 13, 1047410. doi: 10.3389/fpls.2022.1047410 36733604 PMC9887195

[B2] Balestrasse K. B. Benavides M. P. Gallego S. M. Tomaro M. L. (2005). Polyamines and proline are affected by cadmium stress in the nodules and roots of soybean plants. Plant Soil 270, 343–353. doi: 10.1007/s11104-004-1792-030311153

[B3] Bo C. Liu D. Yang J. Ji M. Li Z. Zhu Y. . (2024). Comprehensive in silico characterization of the NAC transcription factor family of Pinellia ternata and functional analysis of PtNAC66 under high-temperature tolerance in transgenic *Arabidopsis thaliana*. Plant Physiol. Biochem. 208, 108539. doi: 10.1016/j.plaphy.2024.108539 38513515

[B4] Bolyen E. Rideout J. R. Dillon M. R. Bokulich N. A. Abnet C. C. Al-Ghalith G. A. . (2019). Reproducible, interactive, scalable and extensible microbiome data science using QIIME 2. Nat. Biotechnol. 37, 852–857. doi: 10.1038/s41587-019-0209-9 31341288 PMC7015180

[B5] Chen Y. Zhou Y. Chao Q. Yang R. Duan Y. Xue J. . (2025). Nitrogen application enhances thermotolerance in Pinellia ternata via modulation of antioxidant capacity. Sci. Hortic. 350, 114343. doi: 10.1016/j.scienta.2025.11434342574925

[B6] Dong J. Zhang L. Ouyang Q. Zhang F. Zheng W. Fernie A. R. . (2026). Abscisic acid alleviates cadmium toxicity in oilseed rape plants by regulating its accumulation, subcellular distribution, and ascorbate-glutathione cycle. Environ. Exp. Bot. 244, 106354. doi: 10.1016/j.envexpbot.2026.10635442574925

[B7] Duan Y. Wang S. Wang R. Zhou Y. Shi A. Liu X. . (2025). Growth, photosynthesis, redox homeostasis, secondary metabolism and metabolomic changes of Pinellia ternata under antibiotic stress: a comparative study of oxytetracycline, norfloxacin and sulfamethazine. Plant Physiol. Biochem. 228, 110215. doi: 10.1016/j.plaphy.2025.110215 40623350

[B8] Fan P. Wu L. Wang Q. Wang Y. Luo H. Song J. . (2023). Physiological and molecular mechanisms of medicinal plants in response to cadmium stress: current status and future perspective. J. Hazard. Mater. 450, 131008. doi: 10.1016/j.jhazmat.2023.131008 36842201

[B9] Guo C. Chen Y. Wang M. Du Y. Wu D. Chu J. . (2022). Exogenous brassinolide improves the antioxidant capacity of Pinellia ternata by enhancing the activity of enzymatic and nonenzymatic defense systems under nonstress conditions. Front. Plant Sci. 13, 917301. doi: 10.3389/fpls.2022.917301 35958199 PMC9358693

[B10] He F. Zhao Q. Huang J. L. Niu M. X. Feng H. C. Shi Y. J. . (2021). The external application of nitrogen alleviates the toxicity of cadmium to poplars via starch and sucrose metabolism. Tree Physiol. 41, 2126–2141. doi: 10.1093/treephys/tpab065 33960381

[B11] Hu Y. He R. Mu X. Zhou Y. Li X. Wang H. . (2025). Cadmium toxicity in plants: from transport to tolerance mechanisms. Plant Signal. Behav. 20, 2544316. doi: 10.1080/15592324.2025.2544316 40843968 PMC12377090

[B12] Ji M. Liu M. Zheng X. Xiao C. Ji Y. Duan Y. . (2025). The Pinellia ternata HD-Zip6 gene positively regulates heat stress tolerance in transgenic Arabidopsis by increasing ROS scavenging activity and NAC019 expression. Plant Stress 16, 100806. doi: 10.1016/j.stress.2025.10080642574925

[B13] Lundberg D. S. Lebeis S. L. Paredes S. H. Yourstone S. Gehring J. Malfatti S. . (2012). Defining the core *Arabidopsis thaliana* root microbiome. Nature 488, 86–90. doi: 10.1038/nature11237 22859206 PMC4074413

[B14] Meena M. Divyanshu K. Kumar S. Swapnil P. Zehra A. Shukla V. . (2019). Regulation of L-proline biosynthesis, signal transduction, transport, accumulation and its vital role in plants during variable environmental conditions. Heliyon. 5, e02952. doi: 10.1016/j.heliyon.2019.e02952 PMC6909094 31872123

[B15] Ng C. W. W. So P. S. Wong J. T. F. Lau S. Y. (2023). Intercropping of Pinellia ternata with Sedum alfredii reduces soil cadmium absorption and improves yield. Environ. pollut. 318, 120930. doi: 10.1016/j.envpol.2022.120930 36565916

[B16] Nossa C. W. Oberdorf W. E. Yang L. Aas J. A. Paster B. J. DeSantis T. Z. . (2010). Design of 16S rRNA gene primers for 454 pyrosequencing of the human foregut microbiome. World J. Gastroenterol. 16, 4135–4145. doi: 10.3748/wjg.v16.i33.4135 20806429 PMC2932916

[B17] Spormann S. Nadais P. Sousa F. Pinto M. Martins M. Sousa B. . (2023). Accumulation of proline in plants under contaminated soils-are we on the same page? Antioxidants 12, 666. doi: 10.3390/antiox12030666 36978914 PMC10045403

[B18] Yang Y. Xiong J. Tao L. Cao Z. Tang W. Zhang J. . (2020). Regulatory mechanisms of nitrogen (N) on cadmium (Cd) uptake and accumulation in plants: a review. Sci. Total Environ. 708, 135186. doi: 10.1016/j.scitotenv.2019.135186 31810697

[B19] Yu Y. Fotopoulos V. Zhou K. Fernie A. R. (2025). Role of the gasotransmitter hydrogen sulfide in plant cadmium stress responses. Trends Plant Sci. 30, 35–53. doi: 10.1016/j.tplants.2024.08.003 39358104

[B20] Zhang C. Liu J. Wang Z. Lu X. Dai L. Tang C. . (2025). Physiological and electrophysiological characteristics of continuously cropped Pinellia ternata and a novel electrical information-based monitoring approach for its cropping obstacles. Front. Plant Sci. 16, 1614478. doi: 10.3389/fpls.2025.1614478 40831724 PMC12358450

[B21] Zhang D. Zhang L. Yuan C. Zhai K. Xia W. Duan Y. . (2024). Brassinolide as a potential rescue agent for Pinellia ternata grown under microplastic conditions: insights into its modulatory role in photosynthesis, redox homeostasis, and AsA-GSH cycling. J. Hazard. Mater. 470, 134116. doi: 10.1016/j.jhazmat.2024.134116 38547753

[B22] Zhang J. Shoaib N. Lin K. Mughal N. Wu X. Sun X. . (2024). Boosting cadmium tolerance in Phoebe zhennan: the synergistic effects of exogenous nitrogen and phosphorus treatments promoting antioxidant defense and root development. Front. Plant Sci. 15, 1340287. doi: 10.3389/fpls.2024.1340287 38362448 PMC10867629

[B23] Zhang L. Shi A. Yuan C. Wang S. Zhou Y. Liu X. . (2025). Effects of microplastics on the toxicity of antibiotics on Pinellia ternata: an exploration of their effects on photosynthesis, oxidative stress homeostasis, secondary metabolism, the AsA-GSH cycle, and metabolomics. Plant Physiol. Biochem. 223, 109883. doi: 10.1016/j.plaphy.2025.109883 40199164

