# Supplementary material for: Exogenous nitrogen mitigation of cadmium stress in Pinellia ternata is associated with the regulation of arginine and proline metabolism
Source: Front Plant Sci. 2026 Sep 21;17:1900436. doi: 10.3389/fpls.2026.1900436 (PMC13634967; doi:10.3389/fpls.2026.1900436)
Supplement: Supplementary file 1 [file SupplementaryFile1.docx]

**Supplementary material**

**Exogenous nitrogen mitigation of cadmium stress in *Pinellia ternata* is associated with the regulation of arginine and proline metabolism**

Zhigui He^1,2^, Yuzhen Dai^2^, Qing Zhang^1,3*^

1. School of Food and Health, Guilin Tourism University, Guilin, 541006, People's Republic of China

2. College of Marine Science and Biotechnology, Guangxi Minzu University, Nanning, 530008, People's Republic of China

3. School of Environmental Science and Engineering, Guilin University of Technology, Guilin 541004, People's Republic of China

*Corresponding author

Dr. Qing Zhang, ORCID iD: 0000-0003-1473-0592.

E-mail: 1291034842@qq.com

Tel: +86773 3690116

**FIGURE S1**. Non-metric multidimensional scaling (NMDS) ordination of rhizosphere bacterial community structure, based on Bray–Curtis dissimilarity, comparing Cd and N+Cd treatments at (A) 3 days, (B) 6 days, and (C) 9 days after treatment. Ellipses represent 95% confidence intervals for each group. Stress values were 0.014, 0.013, and 0.008 for (A), (B), and (C), respectively, indicating that the two-dimensional ordination provides a good representation of the underlying dissimilarity structure.

**FIGURE S2**. Differential abundance of top 10 COG functional categories between N+Cd and Cd treatments at (A) 3 days, (B) 6 days, and (C) 9 days after treatment, as predicted by PICRUSt2. Box plots display the distribution of predicted functional gene abundance across biological replicates. P-values are shown below each comparison. Functional categories discussed in the main text (COG1012, COG0841, COG0491) were identified from the 6-day comparison (B).

**FIGURE S3**. Proposed model of nitrogen-mediated cadmium tolerance in *P. ternata*. Exogenous nitrogen alleviates Cd-induced growth inhibition partly through the regulation of arginine and proline metabolism, antioxidant responses, detoxification-related pathways, and rhizosphere microbial stability. The model was constructed based on the physiological, transcriptomic, metabolomic, and microbial results obtained in this study.

Table S1. Effects of different treatments on growth parameters of *Pinellia ternata*

| Time  (day) | Group | Root length  (cm) | Stem length  (cm) | Stem diameter  (cm) | Leaf area  (cm^2^) |
| --- | --- | --- | --- | --- | --- |
| 3 | CK | 9.67 ± 0.45a | 15.90 ± 0.26a | 0.20 ± 0.02a | 10.42 ± 0.36a |
|  | N | 8.80 ± 0.46ab | 15.43 ± 0.15b | 0.18 ± 0.02ab | 9.06 ± 0.88ab |
|  | Cd | 6.70 ± 0.36c | 15.17 ± 0.12c | 0.17 ± 0.02abc | 6.94 ± 0.80c |
|  | N+Cd | 6.23 ± 0.47c | 15.43 ± 0.15b | 0.20 ± 0.01a | 8.61 ± 0.83b |
| 6 | CK | 11.77 ± 0.42a | 16.97 ± 0.49a | 0.21 ± 0.01a | 12.99 ± 0.50a |
|  | N | 9.37 ± 0.25b | 16.83 ± 0.21a | 0.19 ± 0.02b | 11.27 ± 0.44b |
|  | Cd | 7.00 ± 0.10c | 16.07 ± 0.21b | 0.18 ± 0.01bc | 8.03 ± 0.62c |
|  | N+Cd | 9.17 ± 0.45bd | 16.27 ± 0.35bc | 0.20 ± 0.01abc | 9.09 ± 0.55cd |
| 9 | CK | 12.87 ± 0.21a | 17.30 ±0.36a | 0.24 ± 0.03a | 14.67 ±1.28a |
|  | N | 10.70 ± 0.36b | 17.27 ± 0.15a | 0.20 ± 0.02ab | 11.81 ± 0.76b |
|  | Cd | 7.77 ± 0.42c | 16.63 ± 0.25b | 0.19 ± 0.02bc | 10.67 ± 0.81bc |
|  | N+Cd | 10.20 ± 0.56d | 16.60 ± 0.20b | 0.20 ± 0.01ab | 11.81 ± 0.46b |

Table S2. Effects of different treatments on antioxidant enzyme activities (SOD, POD, CAT) and MDA content in *Pinellia ternata*

| Time  (day) | Group | SOD  (U g^-1^) | POD  (U g^-1^) | CAT  (U g^-1^) | MDA  (nmol mg prot^-1^) |
| --- | --- | --- | --- | --- | --- |
| 3 | CK | 1.17±0.00a | 5684±196a | 86.64±11.68a | 0.28±0.19a |
|  | N | 1.43±0.01b | 2303±213b | 129.97±7.65b | 2.43±1.31b |
|  | Cd | 1.02±0.02c | 1225±84c | 76.45±7.65ac | 13.29±0.97c |
|  | N+Cd | 1.43±0.02b | 1656±113d | 84.10±7.65c | 1.35±0.56bd |
| 6 | CK | 2.08±0.02a | 1535±204a | 56.06±4.41a | 3.51±0.37a |
|  | N | 2.54±0.04b | 6141±441b | 76.45±7.65b | 3.09±0.32ab |
|  | Cd | 2.03±0.05ac | 3560±408c | 63.71±4.41ac | 3.99±0.08ac |
|  | N+Cd | 1.86±0.08d | 882±98d | 48.42±4.42ad | 4.63±0.40d |
| 9 | CK | 2.59±0.06a | 4736±590a | 40.77±4.41a | 3.72±0.74a |
|  | N | 2.02±0.04b | 4181±493ab | 56.06±4.41b | 4.47±0.49ab |
|  | Cd | 1.75±0.09c | 1078±98c | 73.91±4.41c | 4.47±0.19abc |
|  | N+Cd | 1.04±0.05d | 1927±149d | 48.42±8.83d | 6.09±0.50d |

Table S3. Top up- and down-regulated genes in *P. ternata* roots under N+Cd versus Cd treatment at day 3.

| Gene id | NR id | NR annotation | Regulate | log₂FC | q-value |
| --- | --- | --- | --- | --- | --- |
| TRINITY_DN915_c0_g1_i1_16 | -- | -- | up | 13.62 | 2.16E-77 |
| TRINITY_DN9538_c0_g2_i1_4 | -- | -- | up | 15.52 | 1.65E-72 |
| TRINITY_DN1224_c0_g1_i5_16 | -- | -- | up | 14.34 | 4.73E-64 |
| TRINITY_DN3762_c1_g1_i1_14 | KAG5127315.1 | hypothetical protein JHK82_028150 [Glycine max] | up | 14.02 | 1.30E-58 |
| TRINITY_DN533_c0_g1_i1_14 | -- | -- | up | 13.57 | 9.56E-58 |
| TRINITY_DN3453_c0_g1_i1_11 | -- | -- | up | 23.05 | 1.65E-13 |
| TRINITY_DN23580_c0_g1_i2_14 | RKP19415.1 | hypothetical protein ROZALSC1DRAFT_14003 [Rozella allomycis CSF55] | up | 24.45 | 4.44E-15 |
| TRINITY_DN10_c0_g1_i1_11 | TXG46537.1 | hypothetical protein EZV62_027952 [Acer yangbiense] | up | 25.22 | 5.51E-16 |
| TRINITY_DN33869_c0_g1_i1_11 | -- | -- | up | 22.28 | 1.11E-12 |
| TRINITY_DN22203_c0_g1_i1_11 | -- | -- | up | 21.71 | 4.32E-12 |
| TRINITY_DN757_c0_g2_i1_16 | -- | -- | down | -13.73 | 4.07E-59 |
| TRINITY_DN37915_c0_g1_i1_17 | KAI5467175.1 | hypothetical protein BGZ63DRAFT_418895 [Mariannaea sp. PMI_226] | down | -12.90 | 3.47E-52 |
| TRINITY_DN3580_c1_g1_i6_17 | -- | -- | down | -12.91 | 6.99E-52 |
| TRINITY_DN1355_c0_g1_i1_17 | CAG9939003.1 | unnamed protein product [Clonostachys rosea f. rosea IK726] | down | -12.49 | 5.30E-49 |
| TRINITY_DN3292_c0_g1_i1_7 | CAG9938943.1 | unnamed protein product [Clonostachys rosea f. rosea IK726] | down | -12.31 | 1.28E-47 |
| TRINITY_DN36386_c0_g1_i1_7 | KAH8912030.1 | hypothetical protein BR93DRAFT_933117 [Coniochaeta sp. PMI_546] | down | -11.94 | 8.15E-46 |
| TRINITY_DN10667_c0_g1_i1_21 | MQM08764.1 | hypothetical protein [Colocasia esculenta] | down | -11.79 | 2.43E-43 |
| TRINITY_DN22916_c1_g1_i1_13 | AEF98125.1 | cytochrome c oxidase subunit I, partial [Lingulodinium polyedra] | down | -11.79 | 2.43E-43 |
| TRINITY_DN38289_c0_g1_i1_7 | -- | -- | down | -11.69 | 5.79E-43 |
| TRINITY_DN27032_c1_g1_i1_13 | -- | -- | down | -24.42 | 4.78E-15 |

Table S4. Top up- and down-regulated genes in *P. ternata* roots under N+Cd versus Cd treatment at day 6.

| Gene id | NR id | NR annotation | Regulate | log₂FC | q-value |
| --- | --- | --- | --- | --- | --- |
| TRINITY_DN274_c0_g1_i11_6 | -- | -- | up | 13.94 | 1.40E-62 |
| TRINITY_DN32998_c0_g1_i2_3 | -- | -- | up | 15.13 | 7.37E-72 |
| TRINITY_DN1879_c0_g1_i1_18 | -- | -- | up | 14.37 | 1.51E-64 |
| TRINITY_DN1713_c0_g1_i1_13 | -- | -- | up | 14.03 | 2.48E-61 |
| TRINITY_DN126_c0_g1_i3_5 | -- | -- | up | 13.51 | 6.69E-59 |
| TRINITY_DN316_c0_g3_i2_18 | -- | -- | up | 13.69 | 6.17E-59 |
| TRINITY_DN4581_c0_g2_i1_3 | -- | -- | up | 13.22 | 6.45E-55 |
| TRINITY_DN6232_c0_g1_i1_6 | -- | -- | up | 13.19 | 9.63E-55 |
| TRINITY_DN324_c0_g2_i1_3 | -- | -- | up | 12.67 | 5.60E-50 |
| TRINITY_DN1728_c0_g1_i2_21 | -- | -- | up | 21.16 | 1.18E-11 |
| TRINITY_DN45484_c0_g1_i1_24 | ASG92143.1 | polyprotein [Cloning vector pLX-PPV] | down | -15.21 | 1.53E-168 |
| TRINITY_DN300_c0_g1_i2_15 | -- | -- | down | -13.36 | 3.10E-167 |
| TRINITY_DN11542_c0_g1_i1_8 | -- | -- | down | -14.03 | 6.26E-62 |
| TRINITY_DN47522_c0_g1_i1_24 | -- | -- | down | -13.87 | 6.73E-60 |
| TRINITY_DN43063_c0_g1_i1_15 | -- | -- | down | -13.84 | 1.91E-59 |
| TRINITY_DN14352_c1_g1_i1_17 | -- | -- | down | -13.58 | 1.70E-57 |
| TRINITY_DN42588_c0_g1_i1_15 | AJW67423.1 | ATP synthase subunit 6, partial [Fusarium sp. FZ0805] | down | -13.27 | 4.10E-55 |
| TRINITY_DN46676_c0_g1_i1_24 | OAL04138.1 | GroES-like protein [Stagonospora sp. SRC1lsM3a] | down | -13.10 | 7.38E-54 |
| TRINITY_DN6687_c0_g1_i1_20 | -- | -- | down | -12.91 | 2.68E-52 |
| TRINITY_DN15573_c0_g2_i1_13 | -- | -- | down | -12.89 | 5.57E-51 |

Table S5. Top up- and down-regulated genes in *P. ternata* roots under N+Cd versus Cd treatment at day 9.

| Gene id | NR id | NR annotation | Regulate | log₂FC | q-value |
| --- | --- | --- | --- | --- | --- |
| TRINITY_DN5472_c0_g1_i1_2 | ASG92143.1 | polyprotein [Cloning vector pLX-PPV] | up | 17.01 | 1.21E-90 |
| TRINITY_DN3013_c0_g1_i1_19 | ASG92143.1 | polyprotein [Cloning vector pLX-PPV] | up | 15.51 | 1.64E-75 |
| TRINITY_DN6380_c2_g1_i1_22 | -- | -- | up | 14.86 | 4.08E-69 |
| TRINITY_DN1364_c0_g1_i7_16 | MQM20066.1 | hypothetical protein [Colocasia esculenta] | up | 13.38 | 5.04E-56 |
| TRINITY_DN10816_c0_g1_i8_16 | -- | -- | up | 12.66 | 3.35E-50 |
| TRINITY_DN21826_c1_g3_i1_22 | -- | -- | up | 22.18 | 1.19E-12 |
| TRINITY_DN34002_c0_g1_i1_24 | GBG34363.1 | 30S ribosomal protein S19, chloroplastic [Hondaea fermentalgiana] | up | 21.36 | 8.45E-12 |
| TRINITY_DN8255_c0_g3_i1_22 | -- | -- | up | 21.98 | 1.93E-12 |
| TRINITY_DN17190_c0_g2_i1_19 | -- | -- | up | 20.92 | 2.34E-11 |
| TRINITY_DN11652_c0_g2_i1_13 | -- | -- | down | -13.32 | 3.19E-74 |
| TRINITY_DN45484_c0_g1_i1_24 | ASG92143.1 | polyprotein [Cloning vector pLX-PPV] | down | -16.08 | 3.42E-81 |
| TRINITY_DN4077_c0_g1_i1_21 | -- | -- | down | -14.23 | 1.35E-63 |
| TRINITY_DN2266_c2_g1_i1_3 | -- | -- | down | -14.16 | 7.85E-63 |
| TRINITY_DN20495_c0_g1_i1_16 | -- | -- | down | -13.42 | 1.50E-56 |
| TRINITY_DN7561_c0_g4_i1_20 | MQM05873.1 | hypothetical protein [Colocasia esculenta] | down | -13.42 | 1.50E-56 |
| TRINITY_DN971_c0_g2_i1_20 | AAC05126.1 | histone H2B, partial [Malus domestica] | down | -12.95 | 1.05E-52 |
| TRINITY_DN45445_c0_g1_i1_20 | -- | -- | down | -12.92 | 2.26E-51 |
| TRINITY_DN3565_c0_g1_i2_22 | KAG0526282.1 | hypothetical protein BDA96_06G132700 [Sorghum bicolor] | down | -12.92 | 2.26E-51 |
| TRINITY_DN40642_c0_g1_i1_16 | -- | -- | down | -12.76 | 1.74E-50 |

Table S6. NR annotation of key differentially expressed genes involved in the arginine and proline metabolism pathway.

| **No.** | **gene_id** | **NR id** | **NR annotation** |
| --- | --- | --- | --- |
| 1 | TRINITY_DN477_c0_g1_i4_1 | MQM20905.1 | hypothetical protein [Colocasia esculenta] |
| 2 | TRINITY_DN39964_c0_g1_i1_3 | AUF72246.1 | aldehyde dehydrogenase 2B2 [Syntrichia caninervis] |
| 3 | TRINITY_DN34671_c0_g1_i5_6 | MQL76417.1 | hypothetical protein [Colocasia esculenta] |
| 4 | TRINITY_DN48054_c0_g1_i1_9 | GBC26248.2 | nucleotide-binding domain-containing protein [Rhizophagus irregularis DAOM 181602=DAOM 197198] |
| 5 | TRINITY_DN14_c0_g1_i1_11 | -- | -- |
| 6 | TRINITY_DN2556_c0_g1_i1_11 | MQL90620.1 | hypothetical protein [Colocasia esculenta] |
| 7 | TRINITY_DN29393_c0_g1_i2_11 | MQL77241.1 | hypothetical protein [Colocasia esculenta] |
| 8 | TRINITY_DN29697_c0_g1_i1_11 | KAF9157889.1 | Aldehyde dehydrogenase [Mortierella sp. AD010] |
| 9 | TRINITY_DN43572_c0_g1_i1_11 | EXX76042.1 | 1-pyrroline-5-carboxylate dehydrogenase [Rhizophagus irregularis DAOM 197198w] |
| 10 | TRINITY_DN45962_c0_g1_i1_11 | MQL84887.1 | hypothetical protein [Colocasia esculenta] |
| 11 | TRINITY_DN50567_c0_g1_i1_11 | KAF9344354.1 | spermidine resistance protein [Mortierella sp. AD094] |
| 12 | TRINITY_DN55086_c0_g2_i1_11 | KAI3635583.1 | hypothetical protein MIR68_006221 [Amoeboaphelidium protococcarum] |
| 13 | TRINITY_DN23318_c0_g1_i1_14 | KAH7476991.1 | Aldehyde dehydrogenase, mitochondrial [Phytophthora ramorum] |
| 14 | TRINITY_DN11983_c0_g1_i1_15 | CAH1764165.1 | 3530_t:CDS:2 [Claroideoglomus sp. SA101] |
| 15 | TRINITY_DN41838_c0_g1_i1_15 | MQL76417.1 | hypothetical protein [Colocasia esculenta] |
| 16 | TRINITY_DN53274_c0_g1_i1_15 | OAQ36564.1 | FAD-linked oxidoreductase [Linnemannia elongata AG-77] |
| 17 | TRINITY_DN59207_c0_g1_i1_15 | GBC11144.1 | pyrroline-5-carboxylate reductase [Rhizophagus irregularis DAOM 181602=DAOM 197198] |
| 18 | TRINITY_DN19808_c0_g1_i1_17 | KAG6975337.1 | hypothetical protein JG688_00002496 [Phytophthora aleatoria] |
| 19 | TRINITY_DN2051_c0_g1_i12_23 | MQL76417.1 | hypothetical protein [Colocasia esculenta] |
| 20 | TRINITY_DN22142_c0_g1_i11_16 | KAG0479862.1 | hypothetical protein HPP92_010720 [Vanilla planifolia] |
| 21 | TRINITY_DN1601_c0_g1_i3_19 | MQL76417.1 | hypothetical protein [Colocasia esculenta] |
| 22 | TRINITY_DN58536_c0_g1_i1_19 | KAH8548964.1 | spermidine synthase [Umbelopsis sp. PMI_123] |
| 23 | TRINITY_DN60758_c0_g1_i1_19 | CAH1764681.1 | 2325_t:CDS:2, partial [Claroideoglomus sp. SA101] |
| 24 | TRINITY_DN60592_c0_g1_i1_17 | KAH7107026.1 | pyridoxal phosphate-dependent transferase [Auriculariales sp. MPI-PUGE-AT-0066] |
| 25 | TRINITY_DN61182_c0_g1_i1_17 | CAE7764341.1 | unnamed protein product [Symbiodinium sp. KB8] |
| 26 | TRINITY_DN61522_c0_g1_i1_17 | KAI3658415.1 | hypothetical protein MP638_007517 [Amoeboaphelidium occidentale] |
| 27 | TRINITY_DN41519_c0_g1_i4_20 | MQM09459.1 | hypothetical protein [Colocasia esculenta] |
| 28 | TRINITY_DN7861_c0_g1_i3_20 | MQM15185.1 | hypothetical protein [Colocasia esculenta] |
| 29 | TRINITY_DN9856_c1_g1_i6_20 | KAF9600192.1 | hypothetical protein IFM89_005016 [Coptis chinensis] |
| 30 | TRINITY_DN52619_c0_g2_i1_23 | CDH56996.1 | adenosylmethionine decarboxylase [Lichtheimia corymbifera JMRC:FSU:9682] |
| 31 | TRINITY_DN8674_c0_g1_i2_23 | GBC26248.2 | nucleotide-binding domain-containing protein [Rhizophagus irregularis DAOM 181602=DAOM 197198] |

Table S7 KEGG enrichment statistics for arginine and proline metabolism (ko00330) in the N+Cd vs CK comparison at days 3, 6 and 9.

| Day | id | Term | ListHits | ListTotal | PopHits | PopTotal | p-value | q-value | Enrichment_score | geneID |
| --- | --- | --- | --- | --- | --- | --- | --- | --- | --- | --- |
| 3d | ko00330 | Arginine and proline metabolism | 42 | 2903 | 213 | 14207 | 0.630 | 1.000 | 0.965 | TRINITY_DN11429_c0_g1_i2_14;TRINITY_DN13046_c0_g2_i1_7;TRINITY_DN1631_c0_g1_i7_14;TRINITY_DN16796_c0_g1_i1_5;TRINITY_DN16845_c0_g1_i5_19;TRINITY_DN19021_c0_g1_i3_1;TRINITY_DN19871_c1_g1_i12_1;TRINITY_DN2083_c0_g1_i8_5;TRINITY_DN21458_c0_g1_i6_23;TRINITY_DN21958_c0_g1_i1_11;TRINITY_DN23284_c0_g1_i1_11;TRINITY_DN23804_c0_g1_i1_8;TRINITY_DN260_c0_g1_i2_10;TRINITY_DN2704_c0_g1_i8_13;TRINITY_DN27566_c0_g1_i7_20;TRINITY_DN29236_c0_g1_i1_6;TRINITY_DN30241_c0_g2_i1_15;TRINITY_DN31273_c0_g1_i1_2;TRINITY_DN31972_c0_g3_i2_6;TRINITY_DN32062_c0_g1_i2_2;TRINITY_DN32673_c0_g1_i1_8;TRINITY_DN33364_c0_g1_i1_7;TRINITY_DN33498_c0_g1_i1_19;TRINITY_DN34582_c0_g1_i1_23;TRINITY_DN350_c0_g1_i13_10;TRINITY_DN35763_c0_g1_i1_4;TRINITY_DN36040_c0_g1_i1_11;TRINITY_DN3641_c0_g1_i17_24;TRINITY_DN36834_c0_g1_i1_23;TRINITY_DN37686_c0_g2_i1_8;TRINITY_DN37_c0_g1_i11_21;TRINITY_DN38881_c0_g2_i1_16;TRINITY_DN40746_c0_g1_i1_14;TRINITY_DN41712_c0_g1_i1_14;TRINITY_DN42190_c0_g1_i1_12;TRINITY_DN47399_c0_g1_i1_19;TRINITY_DN4968_c1_g1_i2_11;TRINITY_DN49785_c0_g1_i1_23;TRINITY_DN532_c0_g1_i6_24;TRINITY_DN7638_c0_g2_i1_1;TRINITY_DN7880_c0_g1_i33_19;TRINITY_DN7961_c0_g1_i1_23 |
| 6d | ko00330 | Arginine and proline metabolism | 20 | 1642 | 213 | 14207 | 0.867 | 1.000 | 0.812 | TRINITY_DN10502_c0_g1_i12_13;TRINITY_DN11241_c0_g1_i5_6;TRINITY_DN13103_c0_g1_i2_20;TRINITY_DN151_c0_g1_i24_5;TRINITY_DN15373_c0_g1_i1_17;TRINITY_DN16064_c0_g1_i1_5;TRINITY_DN16845_c0_g1_i5_19;TRINITY_DN18484_c0_g1_i1_7;TRINITY_DN19802_c0_g1_i38_18;TRINITY_DN21145_c0_g1_i4_5;TRINITY_DN23901_c0_g1_i1_7;TRINITY_DN2761_c0_g1_i13_19;TRINITY_DN3159_c0_g1_i7_7;TRINITY_DN37686_c0_g2_i1_8;TRINITY_DN37_c0_g1_i11_21;TRINITY_DN3875_c0_g1_i6_19;TRINITY_DN4991_c0_g1_i6_21;TRINITY_DN5596_c0_g2_i1_16;TRINITY_DN7638_c0_g2_i1_1;TRINITY_DN9782_c0_g2_i1_6 |
| 9d | ko00330 | Arginine and proline metabolism | 29 | 1762 | 213 | 14207 | 0.324 | 0.855 | 1.098 | TRINITY_DN10176_c0_g1_i5_1;TRINITY_DN14256_c0_g1_i2_15;TRINITY_DN16645_c0_g1_i3_24;TRINITY_DN16845_c0_g1_i5_19;TRINITY_DN1874_c0_g1_i9_4;TRINITY_DN21145_c0_g1_i4_5;TRINITY_DN21458_c0_g1_i6_23;TRINITY_DN21958_c0_g1_i1_11;TRINITY_DN23284_c0_g1_i1_11;TRINITY_DN260_c0_g1_i2_10;TRINITY_DN2761_c0_g1_i13_19;TRINITY_DN31158_c0_g2_i2_20;TRINITY_DN31972_c0_g3_i2_6;TRINITY_DN33498_c0_g1_i1_19;TRINITY_DN34257_c0_g1_i1_15;TRINITY_DN35763_c0_g1_i1_4;TRINITY_DN36040_c0_g1_i1_11;TRINITY_DN3641_c0_g1_i17_24;TRINITY_DN36834_c0_g1_i1_23;TRINITY_DN37035_c0_g2_i1_20;TRINITY_DN38958_c0_g1_i1_4;TRINITY_DN39020_c0_g1_i1_4;TRINITY_DN40051_c0_g1_i1_4;TRINITY_DN42190_c0_g1_i1_12;TRINITY_DN491_c0_g1_i18_24;TRINITY_DN4968_c1_g1_i2_11;TRINITY_DN5596_c0_g2_i1_16;TRINITY_DN6737_c0_g1_i2_24;TRINITY_DN9782_c0_g2_i1_6 |

Table S8 KEGG enrichment statistics for arginine and proline metabolism (ko00330) in the Cd vs CK comparison at days 3, 6 and 9.

| Day | id | Term | ListHits | ListTotal | PopHits | PopTotal | p-value | q-value | Enrichment_score | geneID |
| --- | --- | --- | --- | --- | --- | --- | --- | --- | --- | --- |
| 3d | ko00330 | Arginine and proline metabolism | 75 | 4292 | 213 | 14207 | 0.065 | 0.430 | 1.166 | TRINITY_DN10176_c0_g1_i5_1;TRINITY_DN10502_c0_g1_i12_13;TRINITY_DN11429_c0_g1_i2_14;TRINITY_DN151_c0_g1_i24_5;TRINITY_DN15373_c0_g1_i1_17;TRINITY_DN1568_c0_g1_i5_3;TRINITY_DN1631_c0_g1_i7_14;TRINITY_DN16778_c0_g1_i1_7;TRINITY_DN16796_c0_g1_i1_5;TRINITY_DN16999_c0_g1_i1_11;TRINITY_DN18484_c0_g1_i1_7;TRINITY_DN19021_c0_g1_i3_1;TRINITY_DN19148_c0_g1_i11_8;TRINITY_DN2083_c0_g1_i8_5;TRINITY_DN21458_c0_g1_i6_23;TRINITY_DN21958_c0_g1_i1_11;TRINITY_DN23284_c0_g1_i1_11;TRINITY_DN23901_c0_g1_i1_7;TRINITY_DN24114_c0_g2_i1_20;TRINITY_DN2453_c0_g1_i1_24;TRINITY_DN25205_c0_g1_i1_24;TRINITY_DN260_c0_g1_i2_10;TRINITY_DN2704_c0_g1_i8_13;TRINITY_DN2761_c0_g1_i13_19;TRINITY_DN2788_c0_g1_i6_17;TRINITY_DN30599_c0_g1_i1_16;TRINITY_DN31158_c0_g2_i2_20;TRINITY_DN3159_c0_g1_i7_7;TRINITY_DN31972_c0_g3_i2_6;TRINITY_DN32062_c0_g1_i2_2;TRINITY_DN32673_c0_g1_i1_8;TRINITY_DN32864_c0_g1_i1_22;TRINITY_DN32879_c0_g1_i1_7;TRINITY_DN33498_c0_g1_i1_19;TRINITY_DN34075_c0_g1_i1_20;TRINITY_DN34582_c0_g1_i1_23;TRINITY_DN35763_c0_g1_i1_4;TRINITY_DN36040_c0_g1_i1_11;TRINITY_DN3641_c0_g1_i17_24;TRINITY_DN36530_c0_g1_i1_16;TRINITY_DN36588_c0_g1_i1_21;TRINITY_DN36834_c0_g1_i1_23;TRINITY_DN37035_c0_g2_i1_20;TRINITY_DN37686_c0_g2_i1_8;TRINITY_DN37_c0_g1_i11_21;TRINITY_DN40051_c0_g1_i1_4;TRINITY_DN40297_c0_g1_i1_3;TRINITY_DN40504_c0_g2_i1_9;TRINITY_DN40746_c0_g1_i1_14;TRINITY_DN4096_c0_g1_i10_24;TRINITY_DN41093_c0_g1_i1_20;TRINITY_DN42190_c0_g1_i1_12;TRINITY_DN4521_c0_g1_i13_22;TRINITY_DN45493_c0_g1_i1_2;TRINITY_DN47278_c0_g1_i1_23;TRINITY_DN47299_c0_g1_i1_23;TRINITY_DN48636_c0_g1_i1_23;TRINITY_DN4968_c1_g1_i2_11;TRINITY_DN5009_c0_g1_i11_20;TRINITY_DN5323_c0_g1_i7_23;TRINITY_DN532_c0_g1_i6_24;TRINITY_DN5546_c0_g1_i6_14;TRINITY_DN5596_c0_g2_i1_16;TRINITY_DN5659_c0_g1_i7_13;TRINITY_DN6737_c0_g1_i2_24;TRINITY_DN6981_c0_g1_i18_13;TRINITY_DN7638_c0_g2_i1_1;TRINITY_DN8003_c0_g1_i13_15;TRINITY_DN8327_c0_g1_i5_9;TRINITY_DN8562_c0_g1_i1_10;TRINITY_DN9183_c0_g1_i12_9;TRINITY_DN925_c0_g1_i11_10;TRINITY_DN941_c0_g1_i20_16;TRINITY_DN9574_c0_g1_i1_3;TRINITY_DN9782_c0_g2_i1_6 |
| 6d | ko00330 | Arginine and proline metabolism | 78 | 4978 | 213 | 14207 | 0.337 | 1.000 | 1.045 | TRINITY_DN10502_c0_g1_i12_13;TRINITY_DN13103_c0_g1_i2_20;TRINITY_DN14256_c0_g1_i2_15;TRINITY_DN151_c0_g1_i24_5;TRINITY_DN15573_c0_g1_i1_23;TRINITY_DN16645_c0_g1_i3_24;TRINITY_DN16796_c0_g1_i1_5;TRINITY_DN16845_c0_g1_i5_19;TRINITY_DN18189_c0_g1_i8_4;TRINITY_DN18312_c0_g1_i1_24;TRINITY_DN18312_c0_g2_i1_24;TRINITY_DN1874_c0_g1_i9_4;TRINITY_DN191_c0_g1_i14_9;TRINITY_DN19802_c0_g1_i38_18;TRINITY_DN19871_c1_g1_i12_1;TRINITY_DN20285_c0_g2_i1_24;TRINITY_DN20599_c0_g1_i1_2;TRINITY_DN21145_c0_g1_i4_5;TRINITY_DN21458_c0_g1_i6_23;TRINITY_DN22249_c0_g1_i11_24;TRINITY_DN23168_c0_g1_i1_24;TRINITY_DN23168_c0_g2_i1_24;TRINITY_DN23901_c0_g1_i1_7;TRINITY_DN23906_c0_g1_i1_24;TRINITY_DN24114_c0_g2_i1_20;TRINITY_DN25205_c0_g1_i1_24;TRINITY_DN26213_c0_g2_i1_15;TRINITY_DN27566_c0_g1_i7_20;TRINITY_DN2761_c0_g1_i13_19;TRINITY_DN2788_c0_g1_i6_17;TRINITY_DN28779_c0_g1_i1_24;TRINITY_DN28797_c0_g1_i1_24;TRINITY_DN29236_c0_g1_i1_6;TRINITY_DN31273_c0_g1_i1_2;TRINITY_DN3159_c0_g1_i7_7;TRINITY_DN31940_c0_g1_i1_23;TRINITY_DN31972_c0_g3_i2_6;TRINITY_DN32673_c0_g1_i1_8;TRINITY_DN34110_c0_g1_i1_24;TRINITY_DN350_c0_g1_i13_10;TRINITY_DN35831_c0_g1_i1_24;TRINITY_DN3641_c0_g1_i17_24;TRINITY_DN37035_c0_g2_i1_20;TRINITY_DN37290_c0_g1_i1_24;TRINITY_DN37686_c0_g2_i1_8;TRINITY_DN38451_c0_g3_i1_23;TRINITY_DN3875_c0_g1_i6_19;TRINITY_DN39159_c0_g1_i1_24;TRINITY_DN39159_c0_g2_i1_24;TRINITY_DN39938_c0_g1_i1_21;TRINITY_DN41093_c0_g1_i1_20;TRINITY_DN41663_c0_g1_i1_12;TRINITY_DN42280_c0_g1_i1_18;TRINITY_DN42609_c0_g1_i1_24;TRINITY_DN43597_c0_g1_i1_24;TRINITY_DN4521_c0_g1_i13_22;TRINITY_DN45347_c0_g1_i1_23;TRINITY_DN46463_c0_g1_i1_20;TRINITY_DN47278_c0_g1_i1_23;TRINITY_DN47299_c0_g1_i1_23;TRINITY_DN47399_c0_g1_i1_19;TRINITY_DN47647_c0_g1_i1_24;TRINITY_DN491_c0_g1_i18_24;TRINITY_DN4968_c1_g1_i2_11;TRINITY_DN4991_c0_g1_i6_21;TRINITY_DN5009_c0_g1_i11_20;TRINITY_DN5323_c0_g1_i7_23;TRINITY_DN5420_c0_g1_i13_3;TRINITY_DN5596_c0_g2_i1_16;TRINITY_DN6469_c0_g1_i5_4;TRINITY_DN6737_c0_g1_i2_24;TRINITY_DN7638_c0_g2_i1_1;TRINITY_DN7961_c0_g1_i1_23;TRINITY_DN8597_c0_g1_i11_3;TRINITY_DN9096_c0_g2_i1_24;TRINITY_DN9574_c0_g1_i1_3;TRINITY_DN9782_c0_g2_i1_6;TRINITY_DN998_c0_g1_i8_6 |
| 9d | ko00330 | Arginine and proline metabolism | 53 | 3312 | 213 | 14207 | 0.317 | 0.976 | 1.067 | TRINITY_DN10176_c0_g1_i5_1;TRINITY_DN11468_c0_g1_i13_10;TRINITY_DN13046_c0_g2_i1_7;TRINITY_DN16796_c0_g1_i1_5;TRINITY_DN18484_c0_g1_i1_7;TRINITY_DN2040_c0_g1_i1_24;TRINITY_DN2062_c0_g1_i11_16;TRINITY_DN21458_c0_g1_i6_23;TRINITY_DN21958_c0_g1_i1_11;TRINITY_DN23284_c0_g1_i1_11;TRINITY_DN23750_c0_g1_i1_7;TRINITY_DN23901_c0_g1_i1_7;TRINITY_DN25205_c0_g1_i1_24;TRINITY_DN260_c0_g1_i2_10;TRINITY_DN2761_c0_g1_i13_19;TRINITY_DN28779_c0_g1_i1_24;TRINITY_DN30241_c0_g2_i1_15;TRINITY_DN31273_c0_g1_i1_2;TRINITY_DN31709_c0_g1_i1_7;TRINITY_DN32062_c0_g1_i2_2;TRINITY_DN32879_c0_g1_i1_7;TRINITY_DN33011_c0_g1_i1_7;TRINITY_DN33364_c0_g1_i1_7;TRINITY_DN33364_c0_g2_i1_7;TRINITY_DN33498_c0_g1_i1_19;TRINITY_DN33674_c0_g1_i1_7;TRINITY_DN33748_c0_g1_i1_23;TRINITY_DN35763_c0_g1_i1_4;TRINITY_DN36305_c0_g1_i1_7;TRINITY_DN3641_c0_g1_i17_24;TRINITY_DN36791_c0_g1_i1_7;TRINITY_DN36834_c0_g1_i1_23;TRINITY_DN37443_c0_g1_i1_7;TRINITY_DN37775_c0_g1_i1_7;TRINITY_DN38451_c0_g3_i1_23;TRINITY_DN39517_c0_g1_i1_19;TRINITY_DN40504_c0_g2_i1_9;TRINITY_DN40746_c0_g1_i1_14;TRINITY_DN41093_c0_g1_i1_20;TRINITY_DN41712_c0_g1_i1_14;TRINITY_DN42069_c0_g1_i1_3;TRINITY_DN42190_c0_g1_i1_12;TRINITY_DN43089_c0_g1_i1_19;TRINITY_DN45102_c0_g1_i1_9;TRINITY_DN45493_c0_g1_i1_2;TRINITY_DN47278_c0_g1_i1_23;TRINITY_DN47299_c0_g1_i1_23;TRINITY_DN47399_c0_g1_i1_19;TRINITY_DN48636_c0_g1_i1_23;TRINITY_DN50058_c0_g1_i1_23;TRINITY_DN50380_c0_g1_i1_23;TRINITY_DN9506_c0_g3_i1_7;TRINITY_DN998_c0_g1_i8_6 |

Table S9 KEGG enrichment statistics for arginine and proline metabolism (ko00330) in the N+Cd vs Cd comparison at days 3, 6 and 9.

| Day | id | Term | ListHits | ListTotal | PopHits | PopTotal | p-value | q-value | Enrichment_score | geneID |
| --- | --- | --- | --- | --- | --- | --- | --- | --- | --- | --- |
| 3d | ko00330 | Arginine and proline metabolism | 57 | 3968 | 213 | 14207 | 0.674 | 1.000 | 0.958133 | TRINITY_DN10176_c0_g1_i5_1;TRINITY_DN11429_c0_g1_i2_14;TRINITY_DN11468_c0_g1_i13_10;TRINITY_DN14417_c0_g1_i1_7;TRINITY_DN14615_c0_g1_i5_12;TRINITY_DN15373_c0_g1_i1_17;TRINITY_DN16064_c0_g1_i1_5;TRINITY_DN1631_c0_g1_i7_14;TRINITY_DN16845_c0_g1_i5_19;TRINITY_DN18286_c0_g1_i2_7;TRINITY_DN19021_c0_g1_i3_1;TRINITY_DN19802_c0_g1_i38_18;TRINITY_DN19871_c1_g1_i12_1;TRINITY_DN20599_c0_g1_i1_2;TRINITY_DN21958_c0_g1_i1_11;TRINITY_DN23284_c0_g1_i1_11;TRINITY_DN23804_c0_g1_i1_8;TRINITY_DN25205_c0_g1_i1_24;TRINITY_DN2704_c0_g1_i8_13;TRINITY_DN2761_c0_g1_i13_19;TRINITY_DN30241_c0_g2_i1_15;TRINITY_DN31709_c0_g1_i1_7;TRINITY_DN31972_c0_g3_i2_6;TRINITY_DN32673_c0_g1_i1_8;TRINITY_DN32879_c0_g1_i1_7;TRINITY_DN33011_c0_g1_i1_7;TRINITY_DN33748_c0_g1_i1_23;TRINITY_DN33808_c1_g1_i1_5;TRINITY_DN33914_c0_g2_i1_7;TRINITY_DN36040_c0_g1_i1_11;TRINITY_DN37686_c0_g2_i1_8;TRINITY_DN37775_c0_g1_i1_7;TRINITY_DN37_c0_g1_i11_21;TRINITY_DN38451_c0_g3_i1_23;TRINITY_DN38881_c0_g2_i1_16;TRINITY_DN39517_c0_g1_i1_19;TRINITY_DN3988_c0_g1_i4_5;TRINITY_DN40504_c0_g2_i1_9;TRINITY_DN40746_c0_g1_i1_14;TRINITY_DN41093_c0_g1_i1_20;TRINITY_DN42190_c0_g1_i1_12;TRINITY_DN43089_c0_g1_i1_19;TRINITY_DN45493_c0_g1_i1_2;TRINITY_DN47278_c0_g1_i1_23;TRINITY_DN47299_c0_g1_i1_23;TRINITY_DN47399_c0_g1_i1_19;TRINITY_DN48636_c0_g1_i1_23;TRINITY_DN4968_c1_g1_i2_11;TRINITY_DN4991_c0_g1_i6_21;TRINITY_DN532_c0_g1_i6_24;TRINITY_DN5596_c0_g2_i1_16;TRINITY_DN6737_c0_g1_i2_24;TRINITY_DN6981_c0_g1_i18_13;TRINITY_DN7880_c0_g1_i33_19;TRINITY_DN7961_c0_g1_i1_23;TRINITY_DN8597_c0_g1_i11_3;TRINITY_DN9506_c0_g3_i1_7 |
| 6d | ko00330 | Arginine and proline metabolism | 15 | 1088 | 213 | 14207 | 0.671 | 1.000 | 0.919571 | TRINITY_DN13046_c0_g2_i1_7;TRINITY_DN15373_c0_g1_i1_17;TRINITY_DN16064_c0_g1_i1_5;TRINITY_DN2083_c0_g1_i8_5;TRINITY_DN21145_c0_g1_i4_5;TRINITY_DN25205_c0_g1_i1_24;TRINITY_DN27566_c0_g1_i7_20;TRINITY_DN2788_c0_g1_i6_17;TRINITY_DN29236_c0_g1_i1_6;TRINITY_DN38451_c0_g3_i1_23;TRINITY_DN39517_c0_g1_i1_19;TRINITY_DN42190_c0_g1_i1_12;TRINITY_DN47399_c0_g1_i1_19;TRINITY_DN48636_c0_g1_i1_23;TRINITY_DN5009_c0_g1_i11_20 |
| 9d | ko00330 | Arginine and proline metabolism | 41 | 3130 | 213 | 14207 | 0.858 | 1.000 | 0.8737 | TRINITY_DN1047_c0_g1_i15_18;TRINITY_DN13103_c0_g1_i2_20;TRINITY_DN14256_c0_g1_i2_15;TRINITY_DN151_c0_g1_i24_5;TRINITY_DN1568_c0_g1_i5_3;TRINITY_DN1631_c0_g1_i7_14;TRINITY_DN16845_c0_g1_i5_19;TRINITY_DN16999_c0_g1_i1_11;TRINITY_DN1794_c1_g1_i5_6;TRINITY_DN18484_c0_g1_i1_7;TRINITY_DN1874_c0_g1_i9_4;TRINITY_DN19021_c0_g1_i3_1;TRINITY_DN191_c0_g1_i14_9;TRINITY_DN19871_c1_g1_i12_1;TRINITY_DN21145_c0_g1_i4_5;TRINITY_DN21958_c0_g1_i1_11;TRINITY_DN260_c0_g1_i2_10;TRINITY_DN2761_c0_g1_i13_19;TRINITY_DN29236_c0_g1_i1_6;TRINITY_DN29290_c0_g1_i1_14;TRINITY_DN31972_c0_g3_i2_6;TRINITY_DN32879_c0_g1_i1_7;TRINITY_DN33498_c0_g1_i1_19;TRINITY_DN3374_c0_g1_i7_17;TRINITY_DN33808_c1_g1_i1_5;TRINITY_DN3641_c0_g1_i17_24;TRINITY_DN38958_c0_g1_i1_4;TRINITY_DN47299_c0_g1_i1_23;TRINITY_DN50058_c0_g1_i1_23;TRINITY_DN5009_c0_g1_i11_20;TRINITY_DN532_c0_g1_i6_24;TRINITY_DN5596_c0_g2_i1_16;TRINITY_DN5659_c0_g1_i7_13;TRINITY_DN6981_c0_g1_i18_13;TRINITY_DN7215_c0_g1_i6_22;TRINITY_DN7638_c0_g2_i1_1;TRINITY_DN7880_c0_g1_i33_19;TRINITY_DN8220_c0_g1_i4_20;TRINITY_DN9183_c0_g1_i12_9;TRINITY_DN925_c0_g1_i11_10;TRINITY_DN941_c0_g1_i20_16 |
